# Supplementary material for: Cdc14 and PP2A Phosphatases Cooperate to Shape Phosphoproteome Dynamics during Mitotic Exit
Source: Cell Rep. 2019 Nov 12;29(7):2105–2119.e4. doi: 10.1016/j.celrep.2019.10.041 (PMC6857435; doi:10.1016/j.celrep.2019.10.041)
Supplement: Document S1 — Figures S1–S7 [file mmc1.pdf]

Cell Reports, Volume *29*

Supplemental Information

Cdc14 and PP2A Phosphatases

Cooperate to Shape Phosphoproteome

Dynamics during Mitotic Exit

Sandra A. Touati, Lorena Hofbauer, Andrew W. Jones, Ambrosius P. Snijders, Gavin Kelly, and Frank Uhlmann

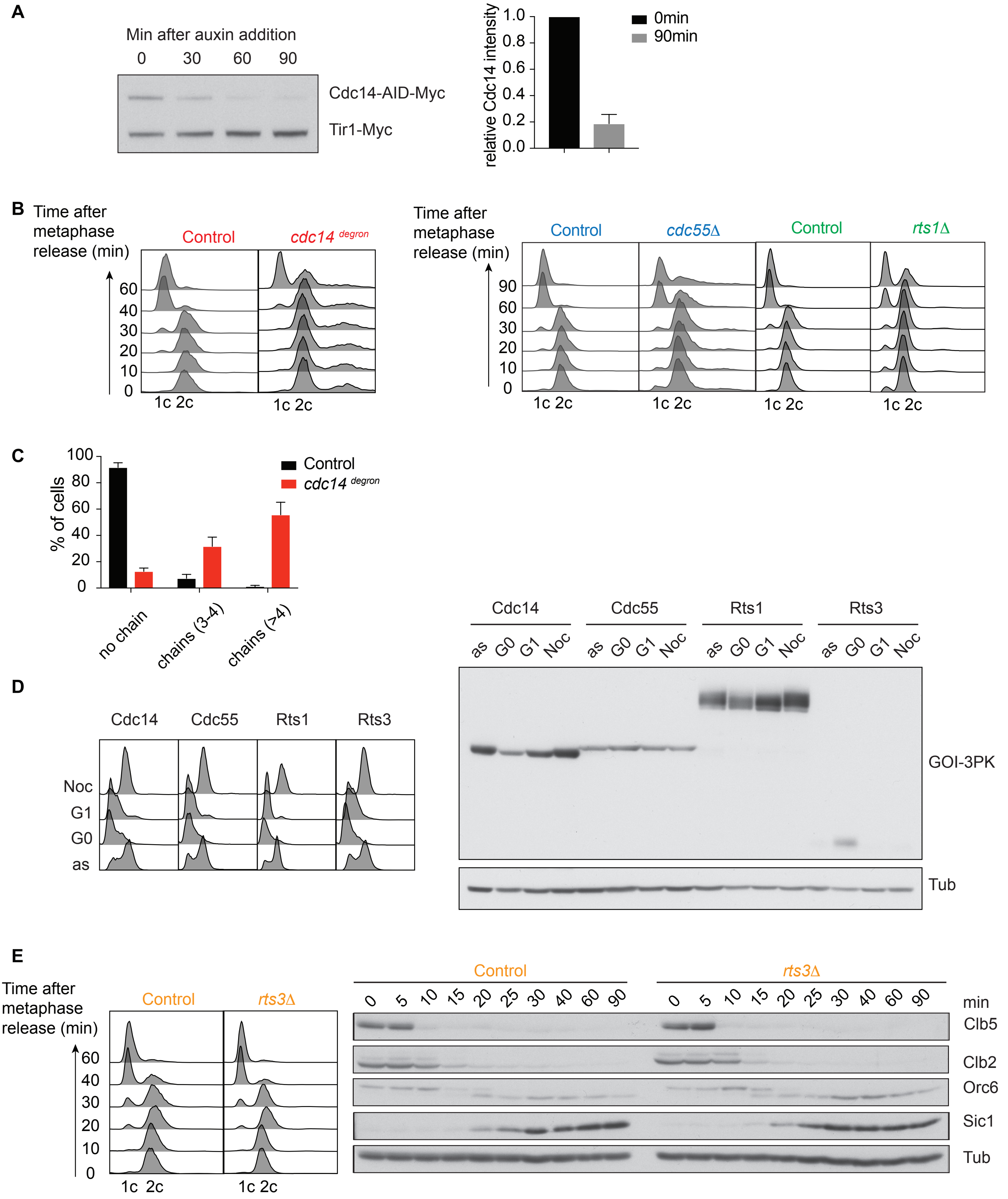

Figure S1. PP2A^{Rts3} Does Not Impact on Mitotic Exit, Related to Figure 1

(A) Cdc14 depletion over time, following auxin addition to a culture of the *cdc14^{degron}* strain, was followed by western blotting (left). Tir1 served as a loading control. Remaining Cdc14 was quantified after 90 minutes (right). The mean and standard deviation from three independent experiments is shown.

(B) FACS analysis of DNA content from a representative experiment shown in Figure 1C.

(C) Cell chain formation was assessed 6 hours following auxin addition to asynchronously proliferating *cdc14^{degron}* cells. The number of connected cell bodies following sonication is depicted. The means and standard deviations from three independent experiments are shown.

(D) Pk epitope-tagged Cdc14, Cdc55, Rts1 or Rts3 was detected in cell extracts from asynchronously growing cells (as), stationary phase cells (G0), following α-factor arrest (G1) and in metaphase arrest following nocodazole (Noc) treatment. Cell cycle stage was confirmed by FACS analysis of DNA content.

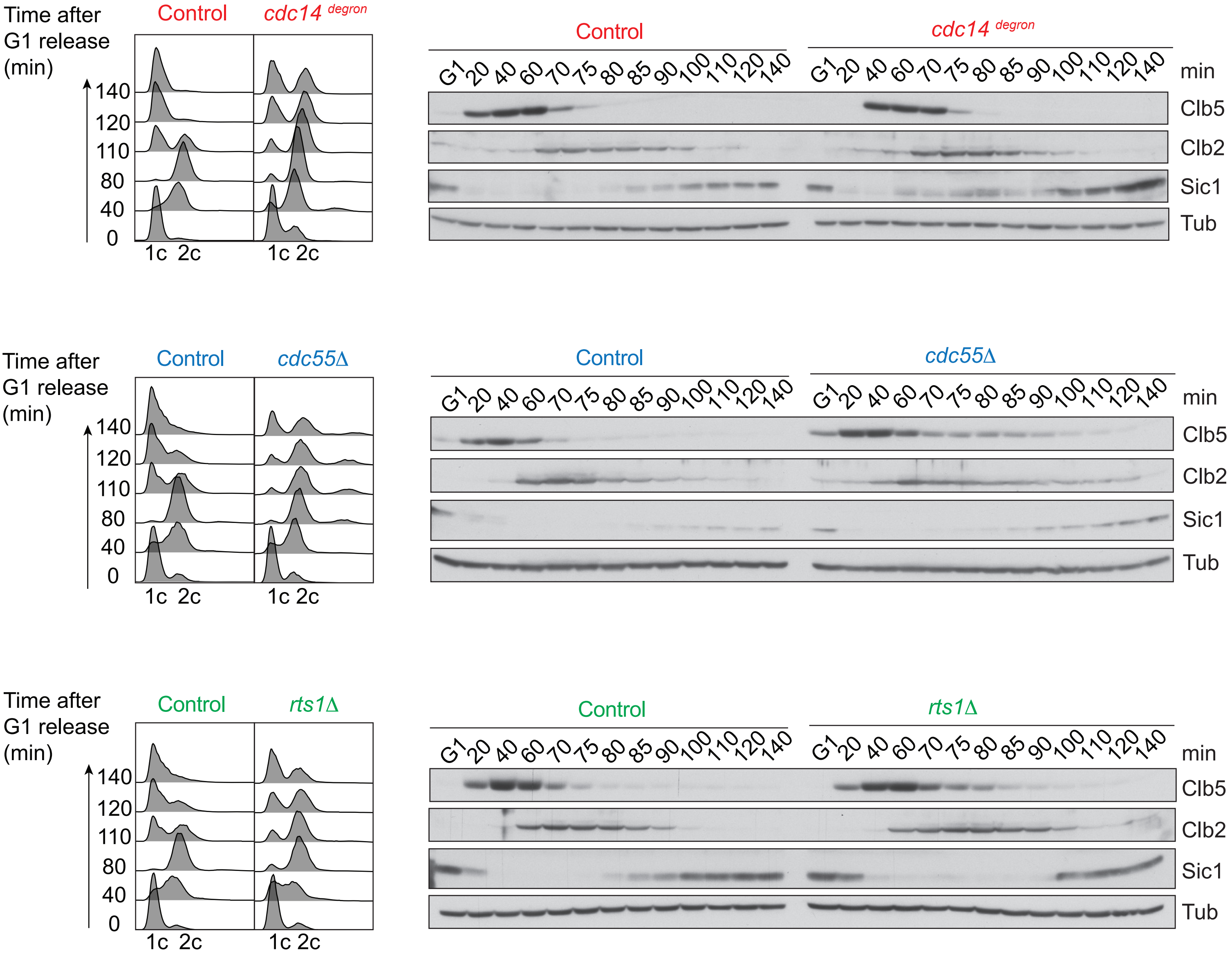

Figure S2. Shared Requirement for Cdc14, PP2A^{Cdc55} and PP2A^{Rts1} for Progression through Mitosis, Related to Figure 1
Control and *cdc14^{degron}* cells were arrested in G1 by α-factor treatment and released to progress synchronously through the cell cycle before being rearrested in G1 by α-factor re-addition. Cell cycle progression was monitored by FACS analysis of DNA content. Protein extracts were prepared at the indicated times and processed for western blotting against the indicated cell cycle proteins. Tubulin served as a loading control. The experiment was repeated with pairs of control and *cdc55*Δ as well as *rts1*Δ cells.

A

		Dataset 1 (*cdc14* ^{degron})	Dataset 2 (*cdc55*Δ)	Dataset 3 (*rts1*Δ)
		Control	Control	Control
sheet 4	(decreasing)	13.1%	10.8%	11.5%
sheet 5	(increasing)	12.2%	14.2%	13.2%
sheet 6	(dip)	1.3%	3.9%	2.4%
sheet 7	(peak)	2.4%	8.7%	4.6%
sheet 8	(flat)	71%	62.4%	68.3%

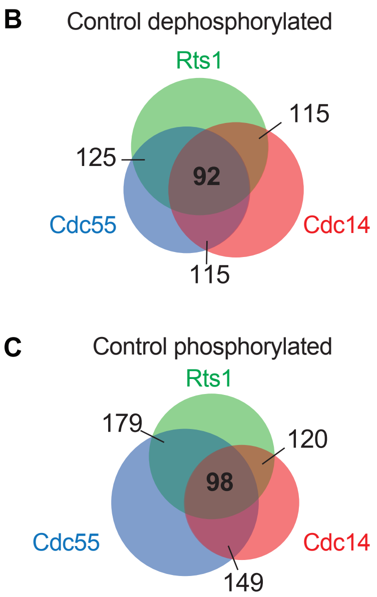

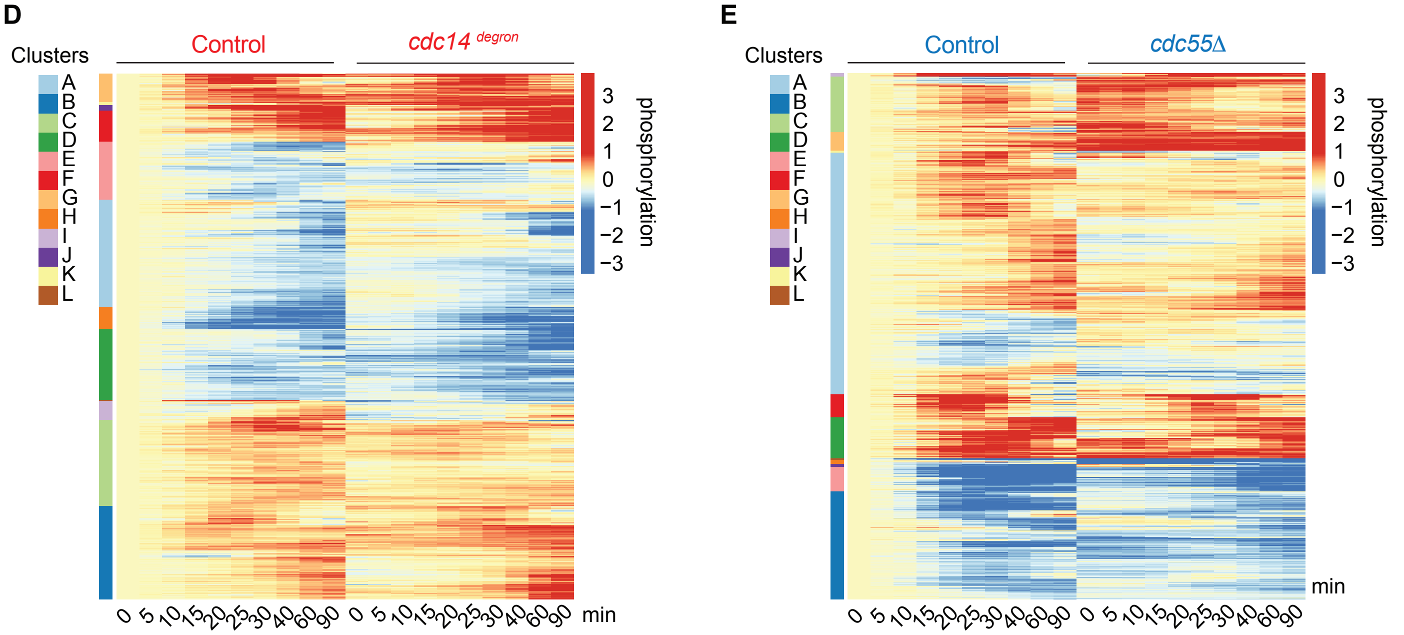

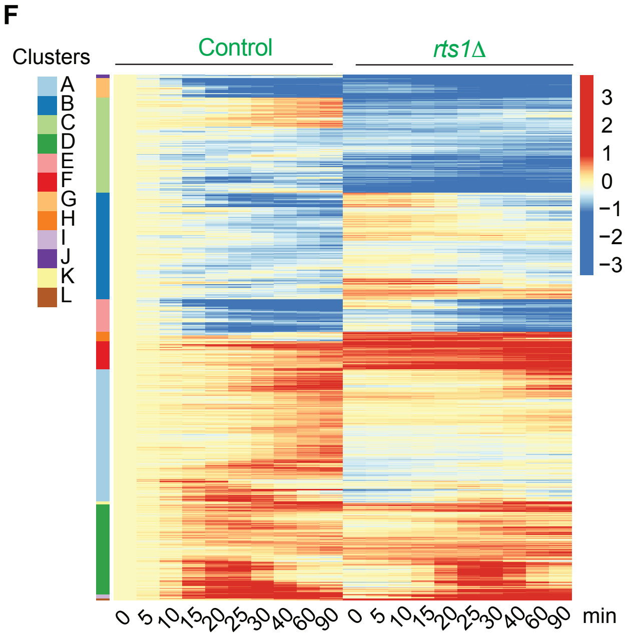

Figure S3. Phosphoproteomics Data Distribution and Heatmap Cluster Analysis, Related to Figure 3

(A) Percentage of phosphosites in each category of each indicated experiment.

(B) Overlap of dephosphorylated phosphosites among the three experimental datasets.

(C) As (B), but overlap of phosphosites that gain in phosphorylation is shown.

(D) Heatmap and cluster analysis of the 691 phosphosites with 1.5 fold phosphorylation changes in either direction in the control strain during mitotic exit in the *cdc14* ^{degron} experiment. Phosphorylation abundance in the control strain was normalized to 0 in metaphase (timepoint 0). Rows were ordered by using 12 hierarchical clusters indicated by different colors. Phosphosite increase (red), decrease (blue), and no change (yellow) are depicted. A natural logarithm scale is used. See also Supplemental Dataset 1 for details of the clusters.

(E) As (D), but 1379 phosphosites from the *cdc55*Δ experimental dataset were analyzed. See also Supplemental Dataset 2.

(F) As (D), but 1522 phosphosites from the *rts1*Δ experimental dataset were analyzed. See also Supplemental Dataset 3.

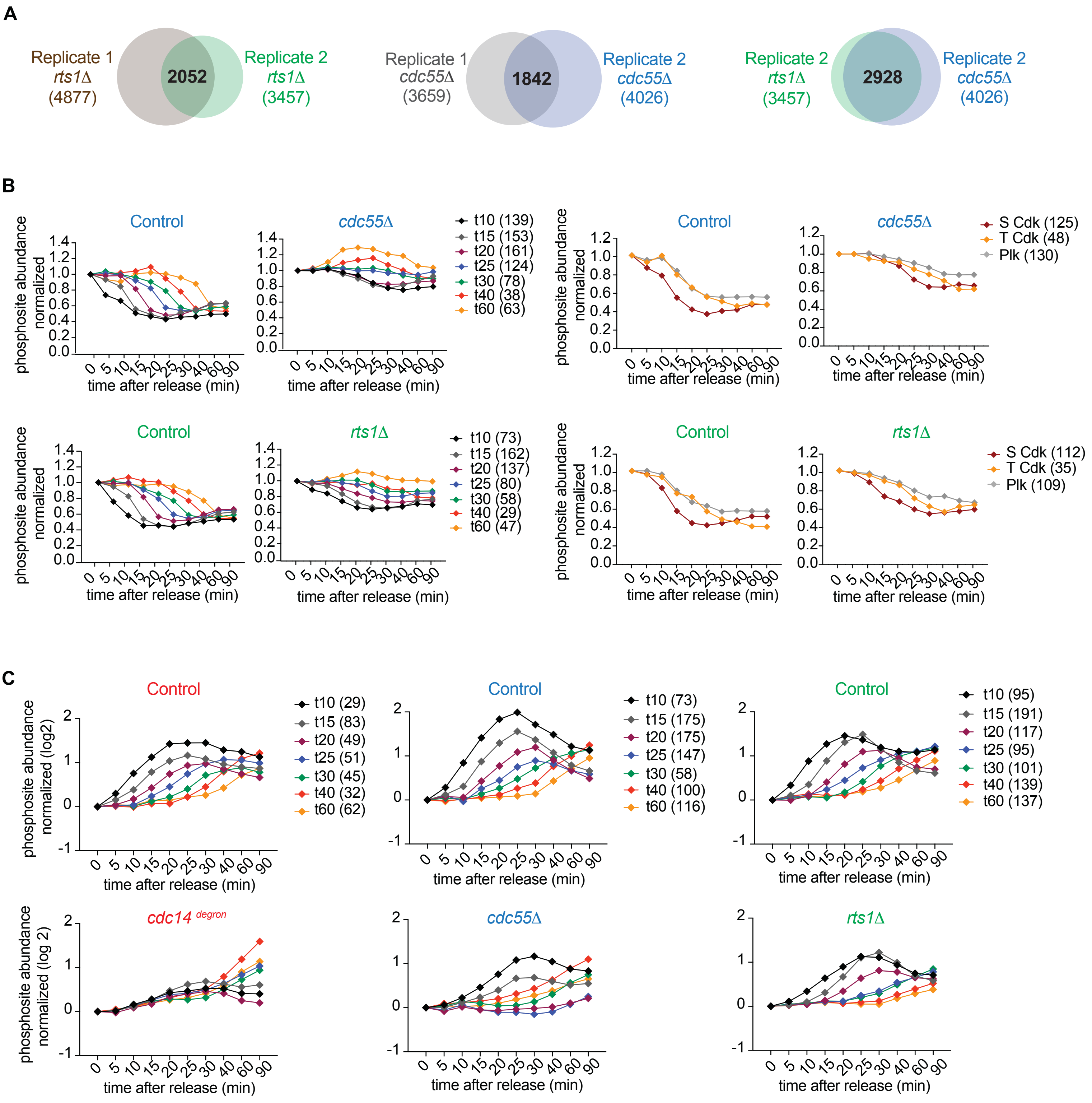

Figure S4. A Biological Replicate and Temporal Order of Phosphorylation Gains During Mitotic Exit, Related to Figure 4

(A) Overlap of phosphosites identified in replicates 1 and 2 of the *cdc55*Δ and *rts1*Δ experimental datasets and between the *cdc55*Δ and *rts1*Δ datasets in replicate 2 which used the same biological control. See also Supplemental Dataset 4.

(B) Normalized mean intensity profiles of phosphosites dephosphorylated during mitotic exit in the control strain of replicate 2, classified according to dephosphorylation timing or kinase consensus motifs, compared to *cdc55*Δ and *rts1*Δ strains as in Figure 4B, C, E and F. See also Supplemental Dataset 4.

(C) Normalized mean intensity profiles of phosphosites that gain abundance during mitotic exit in the control strain in replicate 1. Sites were classified according to the time when their abundance passed the 1.5 fold threshold (top). The same group of phosphosites in the different phosphatase mutant backgrounds are plotted underneath. The number of sites in each group is indicated.

A

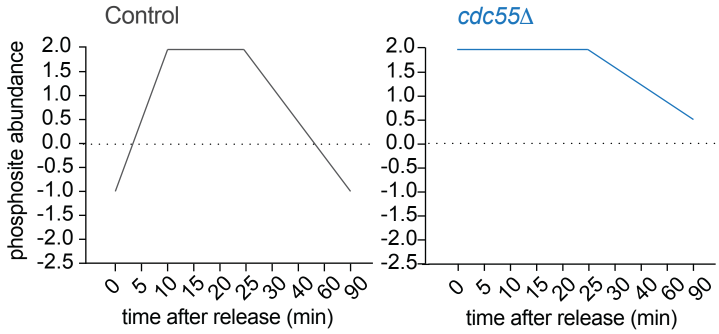

B

Gene names	Positions	Amino acid	Kinase consensus motif
POG1	318	QSANISMANF	Plk
SMI1	355	SQPDTSLQEQ	Plk
YBR138C	241	TKKMVSISDL	
HOF1	424	NQSLSSPSES	Cdk
IDI1	7	TADNNSMPHG	Plk
NET1	288	VDPDKTKQQR	Plk
HOF1	350	QFRNSTPVIR	Cdk / Plk
BUD8	132	PLRNVTPSQN	Cdk / Plk
YCK3	380	KTRNTTPVAT	Cdk / Plk
ADF1	28	KKRSISTAER	
PIB2	346	RKVSETNITN	
NET1	60	RSANVSFTPS	Plk
ERG6	382	TSQEATQ___	Plk
SEC31	1050	QQRVATPLSG	Cdk
PIB2	344	VLRKVSETNI	
KEL1	94	QQRNVSGPST	Plk
KIN1	948	AKREGTPRSI	Cdk / Plk
SYP1	337	LKSKKSSHTL	
NET1	62	ANVSFTPSYF	Cdk
SKG6	167	KYNVRSATPP	
PXL1	44	QRNASTGSLQ	
SPO12	118	QQRFASPTDR	Cdk
TIF4632	880	STKTGSFRNN	
BLI1	109	NNRRQSRTPK	
YCK3	379	SKTRNTTPVA	
ACE2	124	RGLSGTAIFG	
SHE1	287	FSRSTSMNNL	
YMR124W	795	ITSAGSTSGE	
NET1	212	SMRVSTPLAR	Cdk

C

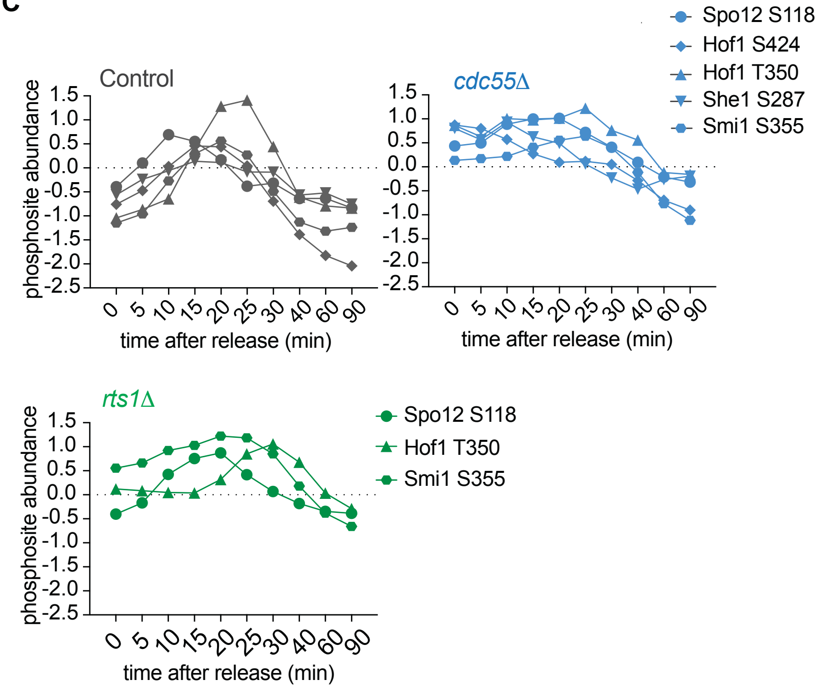

D

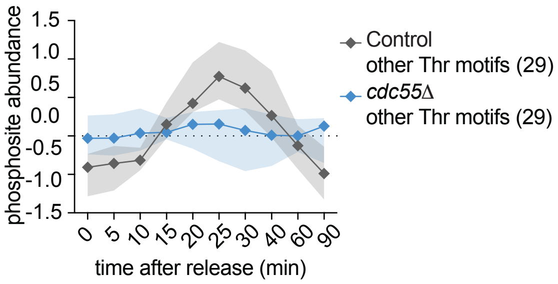

E

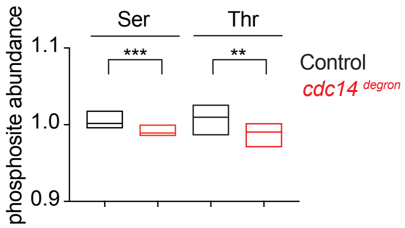

Figure S5. PP2A^{Cdc55} Shapes an Anaphase-Specific Phosphorylation Pattern, Related to Figure 5

(A) Expected profile of an anaphase-specific phosphorylation wave under control of PP2A^{Cdc55}, used as input for correlation analysis.

(B) Table listing phosphosite surroundings and the kinase consensus motif of the 29 identified sites (compare Figure 5C). The phosphorylation site is highlighted in blue.

(C) Example phosphosite profiles of cell cycle substrates controlled by PP2A^{Cdc55} and comparison of lesser control by PP2A^{Rts1}.

(D) Median intensity profile and interquartile range over time of transiently phosphorylated threonine phosphosites that do not adhere to a Cdk or Plk kinase consensus motif.

(E) Median phosphorylation abundance over all 10 time points in control and *cdc14^{degron}* cells, grouped by phosphoacceptor amino acid. 1942 serine and 415 threonine sites entered the analysis. **, p=0.0036; ***, p=0.0006.

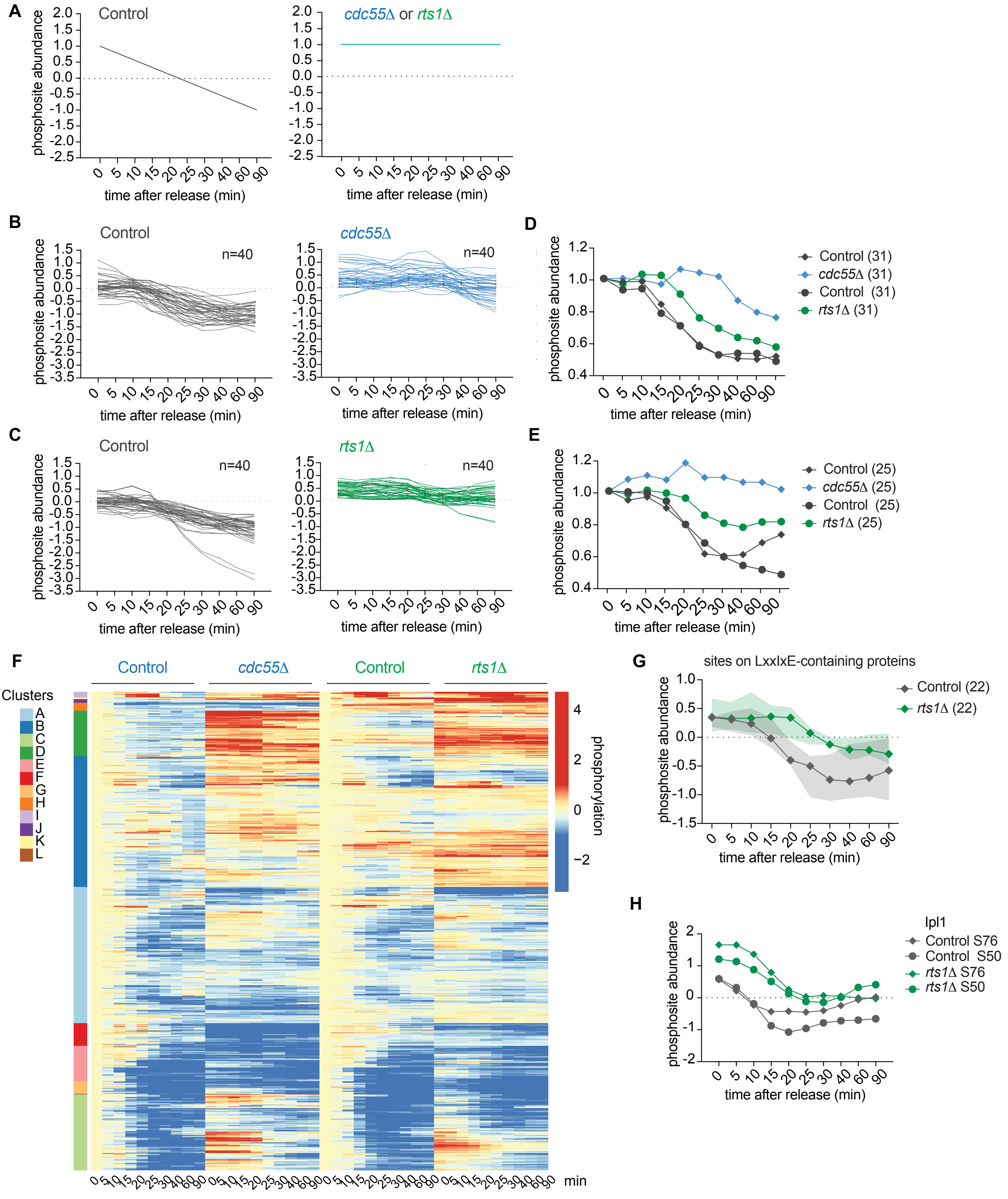

Figure S6. PP2A^{Cdc55} and PP2A^{Rts1} Have Overlapping Substrate Ranges, Related to Figure 6

(A) Expected profiles of PP2A^{Cdc55} or PP2A^{Rts1} substrates, for correlation analysis.

(B) The 40 closest identified PP2A^{Cdc55} substrate profiles. See Supplemental Dataset 2 for a full list of phosphosites.

(C) Normalized median intensity profiles over time of 31 phosphosites in (B) that were also identified in the PP2A^{Rts1} dataset, in control, *cdc55*Δ and *rts1*Δ cells.

(D) The 40 closest identified PP2A^{Rts1} substrate profiles. See Supplemental Dataset 3 for a full list of phosphosites.

(E) Normalized median intensity profiles over time of 25 phosphosites in (D) that were also identified in the PP2A^{Cdc55} dataset, in control, *cdc55*Δ and *rts1*Δ cells.

(F) Heatmap and cluster analysis of the 530 dephosphorylated sites in the control strain in replicate 2 compared to *cdc55*Δ and *rts1*Δ backgrounds, demonstrating related impacts of both phosphatases. See Supplemental Dataset 4 for details of the clusters.

(G) Median intensity profile and interquartile range of 22 phosphosites in 10 proteins harboring a LxxIxE motif in control and PP2A^{Rts1} mutant cells. See Supplemental Dataset 3, sheet 11.

(H) Profile plot of Ipl1 phosphosites in control and *rts1*Δcells.

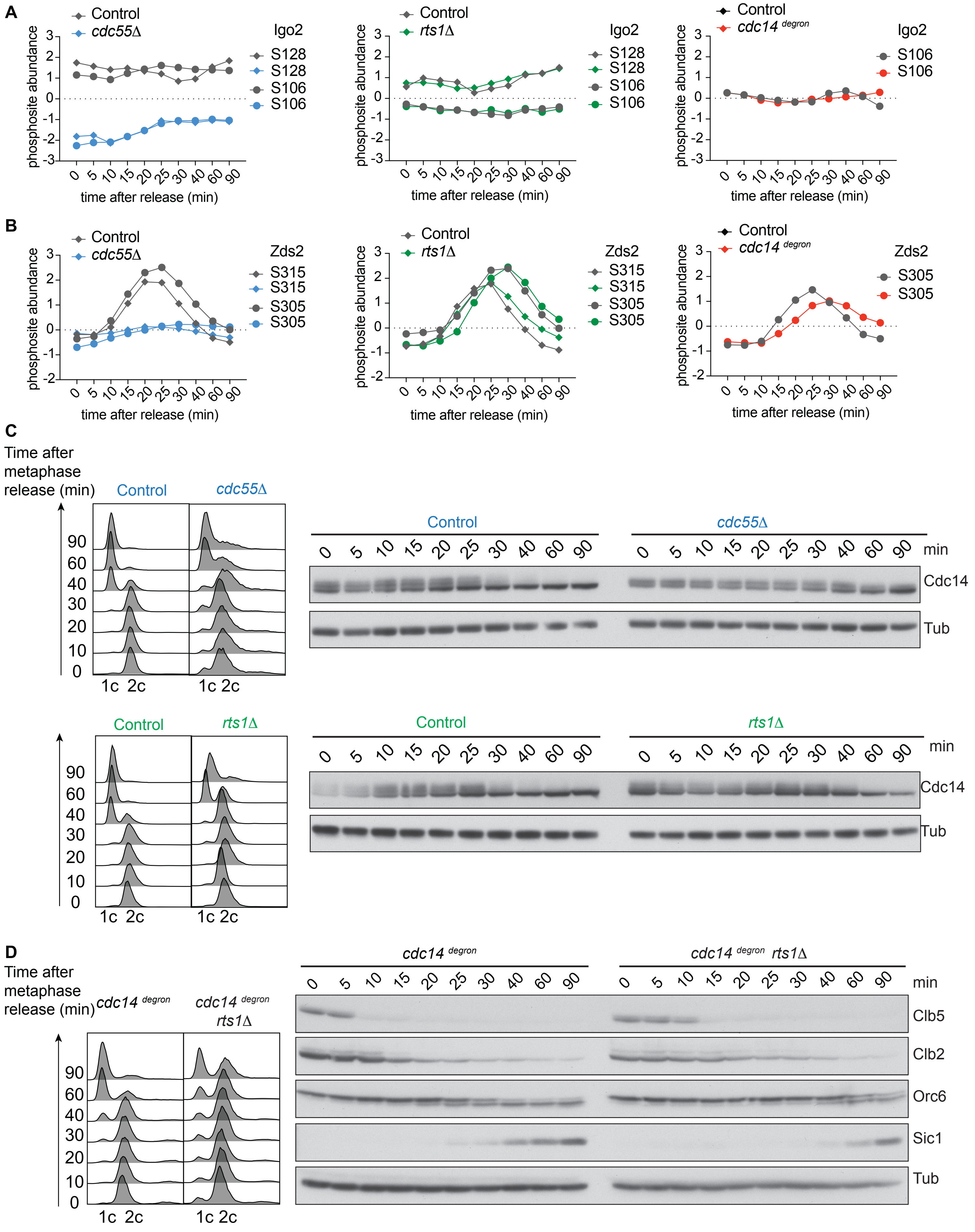

Figure S7. Phosphatase Cooperation during Mitotic Exit, Related to Figure 7

(A) Profile plot of Igo2 phosphosites in control and cdc55Δ cells. Phosphosite profiles are also shown for *rts1*Δ and *cdc14*^{degron} cells.

(B) As (A), but profile plots of Zds2 phosphosites are shown.

(C) Control and cdc55Δ cells (top) or control and *rts1*Δ cells (bottom) were arrested in metaphase and released to progress through mitotic exit as in Figure 1A. Protein extracts were prepared at the indicated times and Cdc14 electrophoretic mobility was monitored by western blotting. FACS analysis of DNA content is shown to confirm synchronous cell cycle progression.

(D) PP2A^{Rts1} acts in synergy with Cdc14. Mitotic exit progression in *cdc14*^{degron} and *cdc14*^{degron} *rts1*Δ cells was compared side-by-side using FACS analysis of DNA content and western blotting against the indicated cell cycle markers. Tubulin served as a loading control.
