# Supplementary material for: Cdc14 and PP2A Phosphatases Cooperate to Shape Phosphoproteome Dynamics during Mitotic Exit
Source: Cell Rep. 2019 Nov 12;29(7):2105–2119.e4. doi: 10.1016/j.celrep.2019.10.041 (PMC6857435; doi:10.1016/j.celrep.2019.10.041)
Supplement: Document S2. Article plus Supplemental Information [file mmc7.pdf]

Cell Reports

Resource

Cdc14 and PP2A Phosphatases Cooperate to Shape Phosphoproteome Dynamics during Mitotic Exit

Graphical Abstract

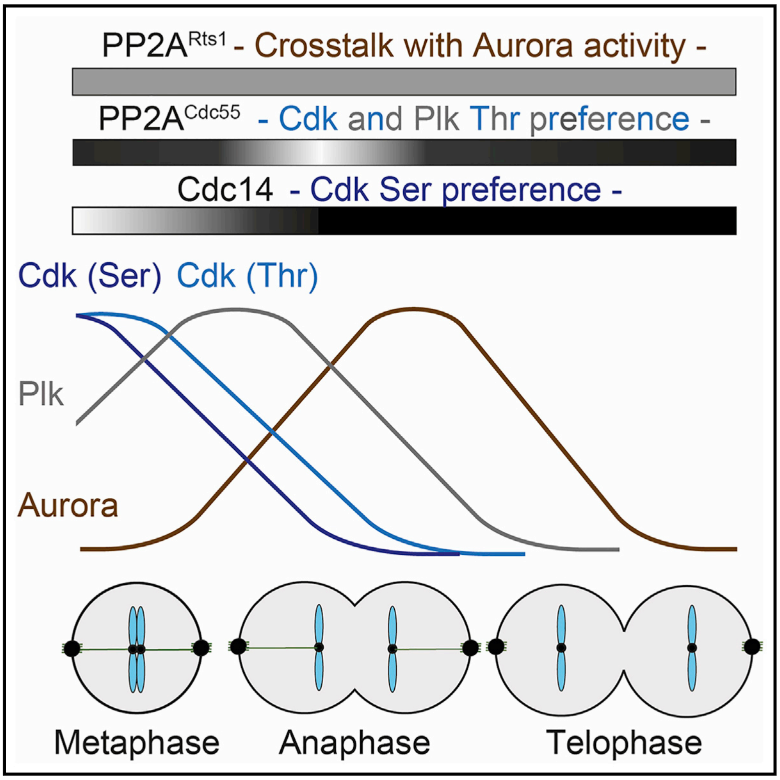

Highlights

- Cdc14, PP2A^{Cdc55}, and PP2A^{Rts1} phosphatases cooperate during budding yeast mitosis
- Cdc14 targets serine Cdk motifs for rapid dephosphorylation
- PP2A^{Cdc55} dephosphorylates Cdk and Plk substrates on threonine residues
- PP2A^{Rts1} displays regulatory crosstalk with Aurora kinase

Authors

Sandra A. Touati, Lorena Hofbauer, Andrew W. Jones, Ambrosius P. Snijders, Gavin Kelly, Frank Uhlmann

Correspondence

sandra.touati@sorbonne-universite.fr (S.A.T.),
frank.uhlmann@crick.ac.uk (F.U.)

In Brief

By using time-resolved phosphoproteome analysis, Touati et al. illuminate the regulatory network that controls temporal order during chromosome segregation and cell division. The work highlights how three different phosphatases cooperate to shape phosphoproteome dynamics at this time, but each also makes unique contributions.

Touati et al., 2019, Cell Reports *29*, 2105–2119
November 12, 2019 © 2019 The Author(s).
https://doi.org/10.1016/j.celrep.2019.10.041

CellPress

Cell Reports
Resource

OPEN ACCESS
CellPress

Cdc14 and PP2A Phosphatases Cooperate to Shape Phosphoproteome Dynamics during Mitotic Exit

Sandra A. Touati,[1,4,*] Lorena Hofbauer,[1,5] Andrew W. Jones,[2] Ambrosius P. Snijders,[2] Gavin Kelly,[3] and Frank Uhlmann[1,6,*]
[1]Chromosome Segregation Laboratory, The Francis Crick Institute, London NW1 1AT, UK
[2]Mass Spectrometry Proteomics Science Technology Platform, The Francis Crick Institute, London NW1 1AT, UK
[3]Bioinformatics & Biostatistics Science Technology Platform, The Francis Crick Institute, London NW1 1AT, UK
[4]Present address: Institut de Biologie Paris Seine, CNRS UMR7622, Sorbonne Université, Paris, France
[5]Present address: Institute of Molecular Pathology, Vienna, Austria
[6]Lead Contact
*Correspondence: sandra.touati@sorbonne-universite.fr (S.A.T.), frank.uhlmann@crick.ac.uk (F.U.)
https://doi.org/10.1016/j.celrep.2019.10.041

SUMMARY

Temporal control over protein phosphorylation and dephosphorylation is crucial for accurate chromosome segregation and for completion of the cell division cycle during exit from mitosis. In budding yeast, the Cdc14 phosphatase is thought to be a major regulator at this time, while in higher eukaryotes PP2A phosphatases take a dominant role. Here, we use time-resolved phosphoproteome analysis in budding yeast to evaluate the respective contributions of Cdc14, $PP2A^{Cdc55}$, and $PP2A^{Rts1}$. This reveals an overlapping requirement for all three phosphatases during mitotic progression. Our time-resolved phosphoproteome resource reveals how Cdc14 instructs the sequential pattern of phosphorylation changes, in part through preferential recognition of serine-based cyclin-dependent kinase (Cdk) substrates. $PP2A^{Cdc55}$ and $PP2A^{Rts1}$ in turn exhibit a broad substrate spectrum with some selectivity for phosphothreonines and a role for $PP2A^{Rts1}$ in sustaining Aurora kinase activity. These results illustrate synergy and coordination between phosphatases as they orchestrate phosphoproteome dynamics during mitotic progression.

INTRODUCTION

Cell-cycle progression is driven by waves of protein phosphorylation and dephosphorylation, mediated by the interplay of cell-cycle kinases and phosphatases (Gelens et al., 2018; Morgan, 2007; Uhlmann et al., 2011). Together with cyclic protein synthesis and degradation, these waves order the sequential events during cell growth and division. The focus of this study lies on the progression through mitosis, when metaphase, anaphase, and cytokinesis have to occur in strict order to avoid cell division failure and consequent aneuploidy. In budding yeast, over 300 proteins are regulated by phosphorylation and dephosphorylation during this time (Touati et al., 2018). The phosphorylation status of a substrate can affect its localization, interactions, and activities, thereby enacting the intricate regulation of mitotic events. Levels of the master cyclin-dependent kinase (Cdk) peak in metaphase, after which anaphase-promoting complex (APC)-mediated cyclin proteolysis initiates downregulation of Cdk activity. Accumulation of a stoichiometric Cdk inhibitor, Sic1, reinforces Cdk downregulation. Additional mitotic kinases include members of the Polo-like kinases (Plks, Cdc5 in budding yeast) and Aurora kinase (Ipl1) that are both also targeted for degradation later on during mitotic exit. In contrast, "nuclear Dbf2-related" (NDR) kinases Mob1-Dbf2 and Mob2-Cbk1 are activated as Cdk activity decreases, thereby promoting late mitosis-specific phosphorylation events that contribute to chromosome segregation and cytokinesis (Afonso et al., 2017; Botchkarev and Haber, 2018; Tamborrini et al., 2018; Weiss, 2012).

Phosphatases are the biological counterforce of kinases. There are an order of magnitude fewer phosphatases encoded in eukaryotic genomes than kinases (Chen et al., 2017). Consequently, phosphatases are thought to be more promiscuous than kinases. However, compared to kinases, their substrate specificities have been less well explored. The Cdc14 phosphatase is thought to provide the major source for protein dephosphorylation during budding yeast mitotic exit (Queralt and Uhlmann, 2008a; Stegmeier and Amon, 2004). It is sequestered by its inhibitor Net1 in the nucleolus for most of the cell cycle. Net1 phosphorylation by a combination of Cdk and Plk releases active Cdc14 at anaphase onset, triggered by separase-mediated downregulation of the $PP2A^{Cdc55}$ phosphatase that counteracts Net1 phosphorylation (Azzam et al., 2004; Queralt et al., 2006). Additional late mitotic kinases probably sustain Net1 phosphorylation and thus Cdc14 activity through mitotic exit. Numerous candidate Cdc14 substrates have been identified using genetic, protein interaction, and phosphoproteomic studies (Bloom and Cross, 2007; Breitkreutz et al., 2010; Kao et al., 2014; Kuilman et al., 2015; Visintin et al., 1998). *In vitro*,

This is an open access article under the CC BY license (http://creativecommons.org/licenses/by/4.0/).

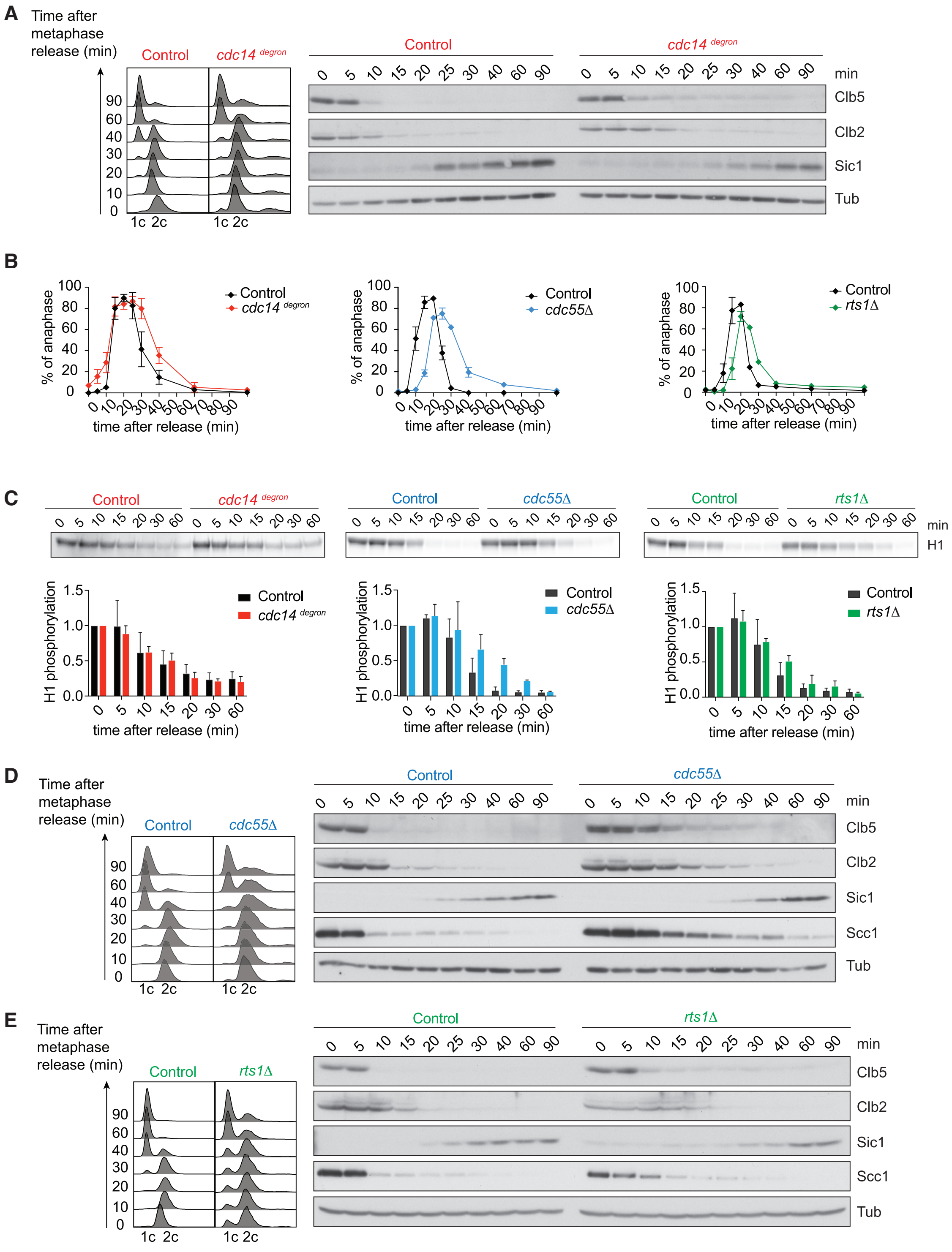

Figure 1. Cdc14, PP2A^{Cdc55}, and PP2A^{Rts1} All Contribute to Mitotic Exit Progression

(A) Control and *cdc14^{degron}* cells were arrested in metaphase by Cdc20 depletion and then released to progress through synchronous mitosis following Cdc20 reinduction. α factor was added to arrest the cells following completion of mitotic exit in G1. Cell-cycle progression was monitored by fluorescence-activated cell sorting (FACS) analysis of DNA content. Protein extracts were prepared at the indicated times and processed for western blotting against the indicated proteins.

(B) The fraction of cells with long anaphase (≥ 2 μm) spindles was scored in aliquots from the experiments in (A), (D), and (E). The mean ± SD of three independent experiments is shown. One hundred cells were scored at each time point in each experiment.

(legend continued on next page)

Cdc14 shows a strong preference for dephosphorylating serine-proline motifs, additionally facilitated by a positive charge at the +3 position (SPx(K/R)) (Bremmer et al., 2012; Eissler et al., 2014). This pattern coincides with the preferred recognition motif for Cdk phosphorylation. The order in which Cdc14 dephosphorylates its substrates during mitotic exit correlates with the relative catalytic efficiencies of Cdc14 and Cdk toward their substrates. Substrates that are better Cdc14 than Cdk substrates are dephosphorylated first (Bouchoux and Uhlmann, 2011). A PxL substrate docking motif contributes to Cdc14 substrate affinity (Kataria et al., 2018).

Cdc14 has important roles in higher eukaryotes, including ciliated sensory cell function, making Cdc14 essential for hearing and male fertility in mouse and humans (Imtiaz et al., 2018; Neitzel et al., 2018). However, organisms aside from budding yeast survive and successfully complete cell division cycles without Cdc14. Instead, a major mitotic exit role has been assigned to members of the abundant PP2A family (Cundell et al., 2016; Manchado et al., 2010; Mochida et al., 2009; Nilsson, 2019; Schmitz et al., 2010). PP2As are heterotrimeric holoenzymes composed of a scaffold and a catalytic subunit, as well as one of a range of regulatory subunits that are thought to provide substrate specificity. Budding yeast PP2A is made up of the scaffold Tpd3; two alternative, but largely interchangeable, catalytic subunits Pph21 or Pph22; as well as one of three regulatory subunits Cdc55 (homolog to human B55), Rts1 (homolog to human B56), and Rts3. PP2A^{Rts1} participates in cell size regulation at the G1/S transition and opposes Aurora kinase to promote chromosome biorientation in mitosis (Peplowska et al., 2014; Zapata et al., 2014). During cytokinesis, PP2A^{Rts1} is involved in the reorganization of septin rings (Dobbelaere et al., 2003). Budding yeast PP2A^{Cdc55} in turn has been invoked in Cdk regulation during interphase (Harvey et al., 2011; Minshull et al., 1996). By the time cells enter mitosis, PP2A^{Cdc55} modulates cell-cycle progression by counteracting phosphorylation of the APC as well as of the Cdc14 inhibitor Net1 (Lianga et al., 2013; Queralt et al., 2006). Proteome-wide surveys uncovered a plethora of PP2A^{Cdc55} substrates. Consistent with its biochemical characteristics, PP2A^{Cdc55} preferentially targets threonine residues, thereby establishing a temporal order of serine before threonine phosphorylation as cells progress from S phase to mitosis (Baro et al., 2018; Godfrey et al., 2017). Little is known about the possible roles of PP2A^{Rts3}. Despite the importance of protein dephosphorylation during mitotic exit, it is not yet known what relative contributions these various phosphatases make at this time.

Here, we use time-resolved phosphoproteome analysis to survey the respective contributions of Cdc14, PP2A^{Cdc55}, and PP2A^{Rts1} to mitotic progression. We take advantage of recent methods that utilize tandem mass tags to allow side-by-side comparison of phosphorylation changes during mitotic progression in the presence or absence of either of these phosphatases. We extend the notion that Cdc14 controls protein dephosphorylation during budding yeast mitotic exit by revealing key contributions of PP2A^{Cdc55} and PP2A^{Rts1}. The three phosphatases act, in part, redundantly, but they also contribute specific and additive roles. Our findings portray mitotic exit as a time when multiple phosphatases cooperate to bring about the phosphorylation changes that orchestrate the completion of faithful chromosome segregation.

RESULTS

Cdc14, PP2A^{Cdc55}, and PP2A^{Rts1} All Contribute to Mitotic Exit Progression

We set out to compare the contributions of three major phosphatases to budding yeast mitotic progression: Cdc14, PP2A^{Cdc55}, and PP2A^{Rts1}. Cdc14 inactivation is usually achieved using temperature-sensitive alleles, leading to a telophase-like, late mitotic arrest with persistent Cdk activity (Visintin et al., 1998). Under these conditions, it is hard to know whether Cdk inactivation failure, compromised Cdc14 function, or both, are the reason for persistent substrate phosphorylation. We therefore engineered a yeast strain in which Cdc14 was fused to an auxin-inducible degron tag (*cdc14^{degron}*) (Nishimura et al., 2009). 90 minutes following auxin addition, Cdc14 levels were reduced to around 20% of wild-type levels (Figure S1A). We now observed cells passing through mitosis, synchronized by depletion and reinduction of the APC coactivator Cdc20. The S-phase cyclin Clb5 was degraded and chromosome segregation, visualized by an elongating anaphase spindle, occurred with equal kinetics in control and Cdc14-depleted cells (Figures 1A and 1B). We then followed overall Cdk activity, measured against the model substrate histone H1 after immunoprecipitation of the Cdk kinase subunit Cdc28. Cdk downregulation was slightly retarded following Cdc14 depletion, but reached completion after a short delay (Figures 1C and S1B). Mitotic cyclin Clb2 degradation, spindle disassembly, and cytokinesis were all delayed in *cdc14^{degron}* cells (Figures 1A and 1B). After longer times, the formation of cell chains as the consequence of cytokinesis failure was observed (Figure S1C). This confirms a role of Cdc14 in late mitosis that involves direct protein dephosphorylation (Kuilman et al., 2015; Powers and Hall, 2017).

We next assessed the contribution of PP2A phosphatases. Of the three PP2A regulatory subunits Cdc55, Rts1, and Rts3, we found the first two expressed at all cell-cycle phases, while Rts3 was preferentially expressed in stationary phase cells (Figure S1D). Consistently, Rts3 made no detectable contribution to mitotic progression (Figure S1E). We therefore turned our attention to PP2A^{Cdc55} and PP2A^{Rts1}.

(C) Cdc28 was immunoprecipitated at the indicated times and its associated kinase activity against histone H1 was measured in control and mutant cells. A representative autoradiogram and quantification of H1 phosphorylation relative to time point 0 of three independent experiments is presented. The means ± SD are shown. See also Figure S1B for the cell-cycle progression analysis by FACS analysis of DNA content

(D) As in (A), but *swe1Δ* and *swe1Δ cdc55Δ* cells were used

(E) As in (A), but *swe1Δ* and *swe1Δ rts1Δ* cells were used.

See also Figure S1 for characterization of the *cdc14^{degron}* allele, an abundance analysis of the three phosphatases, and characterization of PP2A^{Rts3}, as well as Figure S2 for cell-cycle analyses following synchronization in G1.

OPEN
ACCESS
CellPress

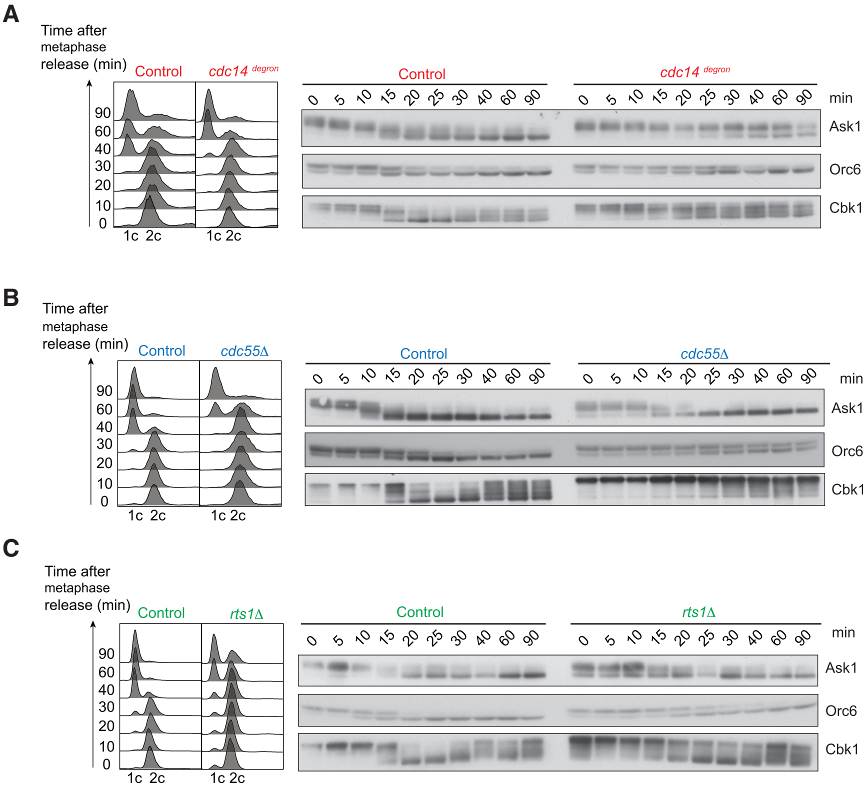

Figure 2. Evidence for Substrate Specificity and Overlap of Cdc14, PP2A^{Cdc55}, and PP2A^{Rts1}

(A) Control and *cdc14^{degron}* cells were arrested and released as described in Figure 1A. Protein extracts were prepared at the indicated times from strains in which Ask1, Orc6, or Cbk1 were fused to an HA epitope tag. A representative FACS analysis of DNA content is shown.

(B) As in (A), but *swe1Δ* and *swe1Δ cdc55Δ* cells were used.

(C) As in (A), but *swe1Δ* and *swe1Δ rts1Δ* cells were used.

Budding yeast cells lacking PP2A^{Cdc55} show gross morphological defects and poor growth due to Cdk inhibitory tyrosine kinase Swe1 activation. For all our experiments with strains lacking Cdc55 or Rts1, we therefore employed a budding yeast strain background lacking Swe1 (*swe1Δ*), which ameliorates many of these defects (Godfrey et al., 2017; Wang and Burke, 1997). We again synchronized cells at the metaphase-to-anaphase transition using Cdc20 depletion and reinduction. This revealed markedly delayed progression through mitosis in cells lacking either PP2A^{Cdc55} or PP2A^{Rts1}. In cells lacking Cdc55, Clb5 degradation and anaphase onset were delayed by 5–10 min (Figures 1D and 1E), maybe due to a role for PP2A in Cdc20 activation (Hein et al., 2017). Spindle disassembly was further delayed and cytokinesis remained inefficient in the absence of PP2A^{Cdc55} or PP2A^{Rts1} (Figure 1B). Cdk downregulation was retarded in the absence of either phosphatase, but it finally reached completion (Figure 1C). This reveals that all three phosphatases—Cdc14, PP2A^{Cdc55}, and PP2A^{Rts1}—are jointly required to promote efficient mitotic progression in budding yeast. We came to the same conclusion when we observed cell-cycle progression using cells synchronized by arrest and release from G1 (Figure S2).

Evidence for Substrate Specificity and Overlap of Cdc14, PP2A^{Cdc55}, and PP2A^{Rts1}

Given that Cdc14, PP2A^{Cdc55}, and PP2A^{Rts1} are all required for timely completion of mitotic exit, we examined if the three phosphatases have overlapping or distinct substrate specificities. The kinetochore protein Ask1, the pre-replicative complex component Orc6, and the NDR-kinase Cbk1 are all thought to be Cdc14 substrates. Their dephosphorylation results in an electrophoretic mobility shift (Bouchoux and Uhlmann, 2011; Brace et al., 2011; Kataria et al., 2018). In the control strains, dephosphorylation of the three substrates occurs around 15–20 min after metaphase release. Following Cdc14 depletion in the *cdc14^{degron}* strain, Ask1 and Orc6 dephosphorylation showed a long delay, while Cbk1 was only ever partially dephosphorylated (Figure 2A). The absence of PP2A^{Cdc55} or PP2A^{Rts1} also delayed Ask1 dephosphorylation, but only for a short time (Figures 2B and 2C). PP2A^{Cdc55} loss delayed Orc6 dephosphorylation to a similar extent as Cdc14 depletion; however, Orc6 dephosphorylation remained unaffected by the absence of PP2A^{Rts1}. Dephosphorylation of Cbk1 in turn was obliterated in the absence of PP2A^{Cdc55}, an effect even greater than following Cdc14 depletion. PP2A^{Rts1} loss only slightly impeded Cbk1 dephosphorylation. These observations suggest that Cdc14, PP2A^{Cdc55}, and PP2A^{Rts1} have overlapping substrate specificities. Their relative contributions vary depending on the individual substrate.

Phosphoproteomics Reveals Phosphatase Contributions to Mitotic Exit

To globally define the *in vivo* substrate ranges of Cdc14, PP2A^{Cdc55}, and PP2A^{Rts1}, we performed time-resolved phosphoproteome analyses. Pairs of strains depleted for, or lacking, the three phosphatases and their respective controls were synchronized as before by Cdc20 depletion and reinduction. Ten samples were collected, spanning from metaphase to G1. Following cell breakage and trypsin digestion, peptides were labeled using 10 isobaric tandem mass tags (TMT10plex). Each pair of two sequential time points received the same label, then sets of five alternating time points of control and mutant were combined to yield two TMT10plex groups (Figure 3A). Phosphopeptides were enriched and liquid chromatography-tandem mass spectrometry (LC-MS/MS) data acquired. The data were filtered to contain only singly phosphorylated peptides, with a phosphosite localization probability score of greater than 0.75. The majority of phosphosites were detected in both of

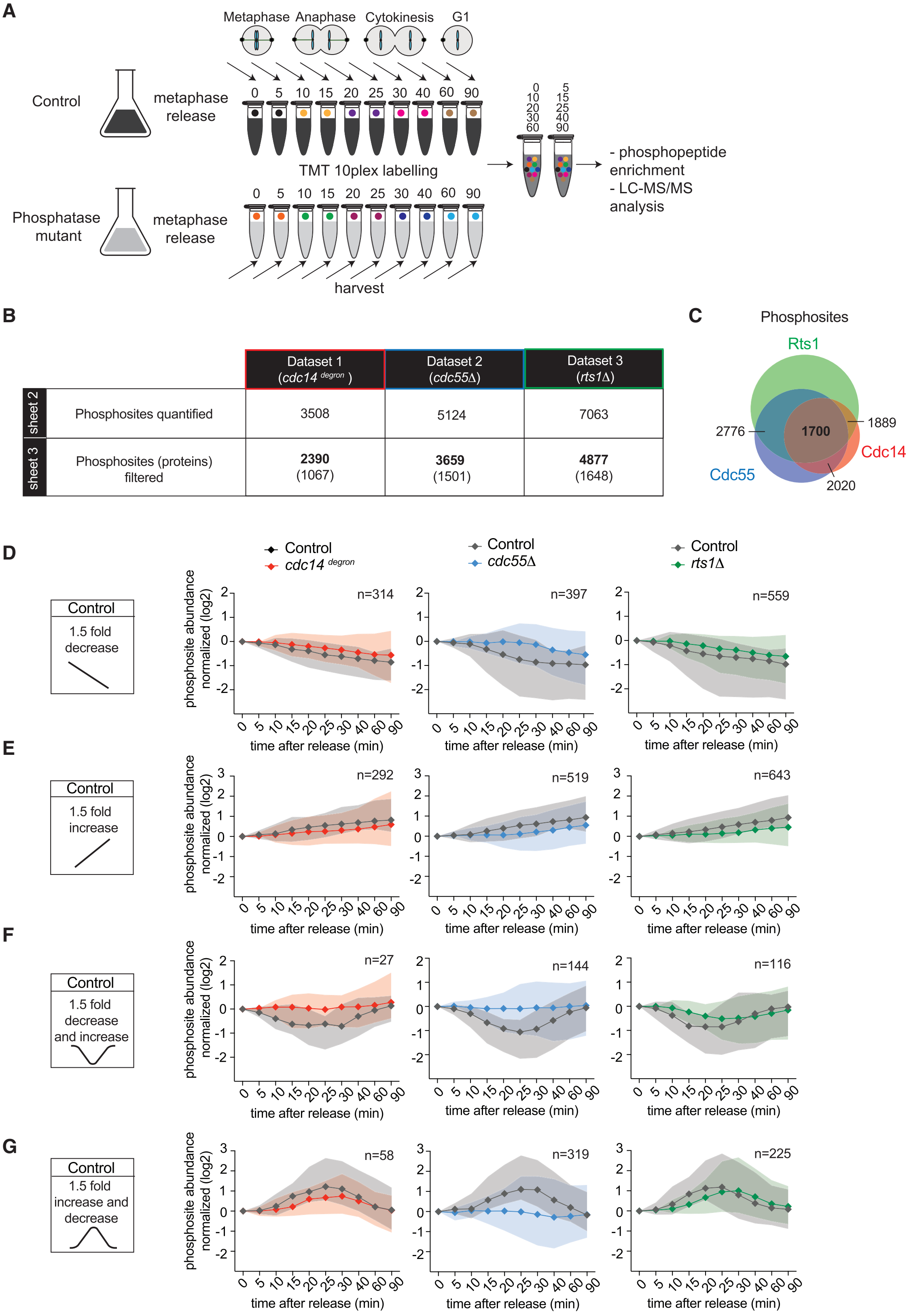

(legend on next page)

the TMT10plex groups, covering all 10 time points. Phosphosites that were detected in only one of the two groups were also included in the analysis and values for the missing alternating time points were filled by imputation (approximately 20% of all values were imputed; see STAR Methods for details). This yielded three datasets that document the impact of the three phosphatases on phosphopeptide dynamics during mitotic progression. They contained 2,390, 3,659, and 4,877 phosphosites in the Cdc14, PP2A^{Cdc55}, and PP2A^{Rts1} datasets, respectively, each representing over 1,000 proteins (Figure 3B; Data S1, S2, and S3). 1,700 phosphosites were common within all three experiments (Figure 3C). We previously found that fewer than 4% of budding yeast proteins change in abundance during mitotic exit (Touati et al., 2018). We therefore expect the majority of phosphosite changes to be the consequence of protein phosphorylation or dephosphorylation.

To categorize phosphosite behavior, abundance at time zero was normalized to 1 (i.e., 0 on a $\log_2$ scale). Phosphosites that decreased 1.5-fold over at least two consecutive time points (i.e., below two-thirds of the time zero value) were classed as dephosphorylated (Figure 3D). Phosphosites that increased in abundance by at least 1.5-fold were considered phosphorylated (Figure 3E). As previously observed (Touati et al., 2018), approximately equal numbers of phosphosites (10%–14% of sites) gained or lost phosphorylation during the course of mitotic exit. In addition, a smaller set of sites showed transient changes. The latter passed the change threshold for at least two consecutive time points but then returned below the threshold for two or more time points (Figures 3F and 3G; see also Data S1, S2, S3, S4, and S5). The distribution of phosphosites between categories and the overlap between the three datasets can be found in Figures S3A–S3C.

For each phosphosite category, we plotted the median and the ranges of the central 90% of sites. The median and ranges of the same sites in the phosphatase mutants were plotted alongside (Figures 3D–3G). This revealed that Cdc14, PP2A^{Cdc55}, and PP2A^{Rts1} all make substantial contributions to the phosphorylation changes seen during mitotic progression. Absence of any of the three phosphatases delayed and attenuated overall protein dephosphorylation during mitotic exit. Equally, protein phosphorylation events were subdued when any of the phosphatases were missing. This suggests that Cdc14, PP2A^{Cdc55}, and PP2A^{Rts1} cooperate during protein dephosphorylation, as well as probably during activation of mitotic kinases that bring about late mitotic phosphorylation. Among the shared contributions of the three phosphatases, we noted a particularly important role of PP2A^{Cdc55} in enacting transient phosphorylation or dephosphorylation events (Figures 3F and 3G), which we will further explore below.

To analyze phosphosite behavior in more detail, we performed unsupervised hierarchical clustering of all the phosphosites that displayed a greater than 1.5-fold change (Figures S3D–S3F). This confirmed grouping of transient and lasting changes. While the dendrogram shows fine detail of phosphosite behavior, we will limit our current analysis to the two broad categories of transient or lasting phosphorylation changes (Figure 3D–3G).

Cdc14 Enacts Dephosphorylation Order by Targeting the Cdk Signature Motif

Cdc14 is thought to sequentially dephosphorylate substrates that play roles during the consecutive phases of mitotic exit (Jin et al., 2008; Bouchoux and Uhlmann, 2011; Kuilman et al., 2015). Our phosphoproteome survey allowed us to come to a global assessment of dephosphorylation timings. We classified all dephosphorylated sites according to their time of dephosphorylation, i.e., when they first passed the 1.5-fold threshold, and plotted their mean dephosphorylation timing (Figure 4A). This confirmed sequential protein dephosphorylation throughout mitotic progression. Plotting the behavior of the phosphosites in the three phosphatase mutants revealed a dramatic impact of Cdc14 depletion. The distinction between early and late dephosphorylation vanished and most phosphosites were concomitantly dephosphorylated with intermediate timing. The dynamic range of dephosphorylation timings was also reduced in cells lacking PP2A^{Cdc55}, although the order of dephosphorylation was maintained (Figure 4B). PP2A^{Rts1} had the least impact on protein dephosphorylation timing (Figure 4C). This suggests that Cdc14 plays a key role in controlling sequential protein dephosphorylation during mitotic exit.

In the above comparison, the dynamic range of protein dephosphorylation was smaller in the control strain for Cdc14 depletion, compared to the control strains for cells lacking

Figure 3. Phosphoproteomics Reveals Phosphatase Contributions to Mitotic Exit

(A) Schematic of the experiment using TMT10plex to analyze phosphoproteome changes in a time-resolved manner. Two TMT10plex sets were used to generate each experimental dataset. Each isobaric mass tag is represented by a different color. One set was used to label the control and the mutant samples at times 0, 10, 20, 30, and 60 min. The other set was used to label samples at 5, 15, 25, 40, and 90 min. After mixing, phosphopeptide enrichment and liquid chromatography-tandem mass spectrometry (LC-MS/MS) were performed.

(B) Table summarizing the three experimental datasets. See Data S1, S2, and S3 for all phosphosite intensities in the *cdc14^{degron}*, *cdc55Δ*, and *rts1Δ* datasets, respectively. The numbers before and after filtration for singly phosphorylated sites with a localization probability score of > 0.75 are indicated. The number of represented proteins is indicated in parentheses.

(C) Overlap of phosphosites between the three experimental datasets.

(D) Normalized median intensity profile and distribution of the central 90% of the phosphosites that undergo a 1.5-fold decrease in phosphorylation abundance through mitotic exit in the control. The respective controls are in black, *cdc14^{degron}* in red, *cdc55Δ* in blue, and *rts1Δ* in green. The number of phosphosites in each category is indicated.

(E) As in (D), but phosphosites that show a 1.5-fold abundance increase in the control are shown.

(F) As in (D), but phosphosites undergoing a transient 1.5-fold decrease in the control are shown.

(G) As in (D), but phosphosites undergoing a transient 1.5-fold increase in the control are shown.

See also Figure S3 for further details of the phosphoproteome analysis, including heatmap and cluster analysis, as well as Data S1, S2, and S3 (sheets 2–8) for a full list of the phosphosites.

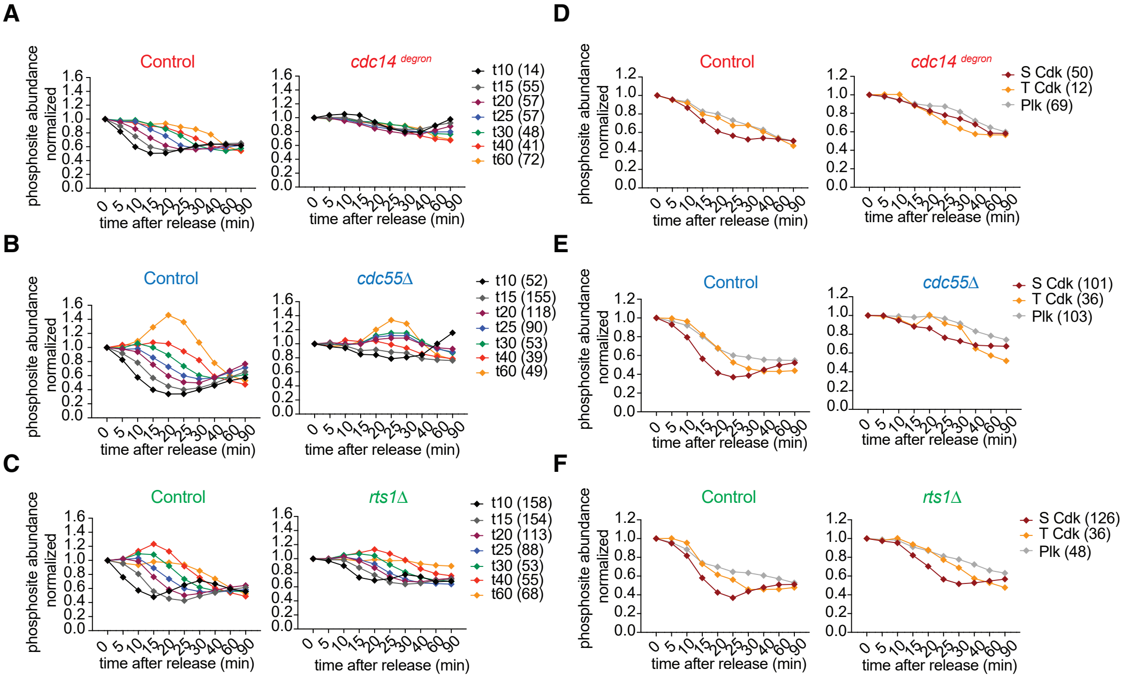

Figure 4. Cdc14 Enacts Dephosphorylation Order by Targeting the Cdk Signature Motif

(A) Normalized mean intensity profiles of phosphosites dephosphorylated during mitotic exit in the control strain. Sites were classified according to dephosphorylation timing (left). The same group of phosphosites in the *cdc14^{degron}* strain are plotted (right). The number of sites in each group is indicated.

(B) As in (A), but comparing control and *cdc55Δ* datasets.

(C) As in (A), but comparing control and *rts1Δ* datasets.

(D) Normalized median intensity profiles over time of phosphosites dephosphorylated in the control strain that adhere to the three indicated kinase consensus motifs (left). The same phosphosites in the *cdc14^{degron}* strain are plotted (right). The number of sites in each group is indicated.

(E) As in (D), but comparing the control and *cdc55Δ* datasets.

(F) As in (D), but comparing the control and *rts1Δ* datasets.

See also Figure S4 and Data S4 for a biological replicate of this analysis and for the temporal analysis of protein phosphorylation.

PP2A^{Cdc55} and PP2A^{Rts1}. This might have contributed to the apparently greater effect seen upon Cdc14 depletion. However, note that Cdc14 depletion is incomplete under our conditions, so that this experiment provides a lower estimate for the contribution of Cdc14. To further test the robustness of our results, we confirmed the impact of PP2A^{Cdc55} and to a lesser extent PP2A^{Rts1} on dephosphorylation dynamics in a biological replicate analysis (Figures S4A–S4C; Data S4). This repeat analysis further extended the number of phosphosites covered in our analysis and reconfirmed several of our key observations (Figures S4A–S4C).

Cdc14 prefers a full Cdk consensus motif (S/T)Px(K/R) for dephosphorylation, with a marked *in vitro* preference for serine over threonine residues (Bremmer et al., 2012; Kuilman et al., 2015; Powers and Hall, 2017). To address whether this specificity shapes the global dephosphorylation order, we classified phosphosites by their kinase consensus motif signatures. Plotting the median dephosphorylation timing of full Cdk consensus motifs confirmed that SPx(K/R) sites are dephosphorylated before TPx(K/R) sites and that both in turn are dephosphorylated before (D/E/N)x(S/T) Plk consensus motifs (Touati et al., 2018). Cdc14 depletion caused loss of this order, with serine-directed sites losing their early dephosphorylation advantage (Figure 4D). Again, PP2A^{Cdc55} and PP2A^{Rts1} had a smaller impact (Figures 4E, 4F, and S4B). This suggests that Cdc14 controls dephosphorylation timing in part by its selectivity for serine-based Cdk phosphomotifs.

Cdc14 controls the activation of several late mitotic kinases (Brace et al., 2011; Jaspersen and Morgan, 2000). Consistently, the temporal order of mitotic phosphorylation events was also greatly impaired following Cdc14 depletion. PP2A^{Cdc55} also participated in setting up the correct phosphorylation order, while PP2A^{Rts1} made only a small contribution (Figure S4C). Together, the three phosphatases provide key input to the accurate order of phosphoproteome changes seen during mitotic exit.

PP2A^{Cdc55} Shapes a Transient Anaphase-Specific Phosphorylation Pattern

Anaphase-specific attenuation of PP2A^{Cdc55} activity, by a mechanism that involves separase and the PP2A^{Cdc55} interactors Zds1 and Zds2 (Queralt et al., 2006; Queralt and Uhlmann, 2008b; Játiva et al., 2019), permits Cdk and Plk phosphorylation of Cdc14's stoichiometric inhibitor Net1, thereby releasing Cdc14 inhibition. The high temporal resolution of our resource allowed us to follow the phosphorylation patterns of 21 Net1

phosphosites. Nine of these fell into the transiently phosphorylated category, showing characteristic anaphase-specific hyperphosphorylation, followed by dephosphorylation when Net1 resequesters Cdc14 at the end of mitotic exit. In cells lacking PP2A^{Cdc55}, six of these sites were prematurely phosphorylated, likely explaining precocious Cdc14 release (Figure 5A). The three other sites were only mildly affected by the absence of PP2A^{Cdc55} (Figure 5B). Cdc14 and PP2A^{Rts1} impacted Net1 phosphorylation only to a lower degree.

We next asked whether the anaphase-specific phosphorylation pattern is unique to Net1, or whether other proteins might be similarly regulated. We designed an expected phosphorylation profile based on the six PP2A^{Cdc55}-regulated Net1 phosphosites and searched for sites with a similar behavior (Figure S5A). This identified 29 phosphosites with anaphase-specific phosphorylation in control cells and premature phosphorylation in the absence of PP2A^{Cdc55} (Figures 5C and S5B). Among these were known mitotic cell-cycle regulators, including the Cdc14 Early Anaphase Release (FEAR)-network component Spo12, cytokinesis regulator Hof1, mitotic spindle protein She1, as well as the cell-wall assembly factor Smi1. The Spo12 and Hof1 sites are previously known Cdk-dependent phosphosites important for mitotic regulation (Meitinger et al., 2011; Tomson et al., 2009). These sites were impacted by PP2A^{Cdc55} and to a lower degree by PP2A^{Rts1} (Figure S5C). This suggests that PP2A^{Cdc55} downregulation at anaphase onset provides an opportunity to shape an anaphase-specific phosphorylation pattern that is used to regulate several biological pathways.

To gain insight into how these anaphase-specific targets are specified, we considered the sequence context of the affected sites. We noticed that five out of the six regulated Net1 phosphosites carry a Cdk or Plk phosphomotif signature, while the three unaffected Net1 sites did not (Figures 5A and 5B). Many of the 29 additionally identified phosphosites also adhere to a Cdk or Plk kinase consensus motif (Figure S5B). To explore this further, we generated a sequence logo of these sites. Enrichment analysis revealed a threonine-directed Plk consensus motif NxT (Figure 5D). We now turned to all 319 transiently phosphorylated sites that we had initially identified (Figure 3F). These contained 12 threonine Plk sites, 16 threonine Cdk sites, and 29 additional threonine-directed sites. Plotting their median and interquartile ranges revealed a general trend that metaphase phosphorylation is counteracted by PP2A^{Cdc55} (Figures 5E and S5D). This demonstrates how a time-resolved phosphoproteome resource allows the characterization of an anaphase-specific phosphorylation wave.

Lastly, we tested whether we can detect a global phosphoramino-acid preference of PP2A^{Cdc55} during mitotic exit. This revealed a substantial threonine preference irrespective of sequence context and mitotic exit stage, consistent with previous observations during interphase (Godfrey et al., 2017). *In vivo* threonine specificity was to a lesser extent also observed in the case of PP2A^{Rts1} but not in the case of Cdc14 (Figures 5F and S5E).

PP2A^{Cdc55} and PP2A^{Rts1} Target an Overlapping Substrate Range

To identify additional dephosphorylation targets of PP2A^{Cdc55} and PP2A^{Rts1}, we looked for phosphosites that are dephosphorylated in control cells but remain stable without either PP2A^{Cdc55} or PP2A^{Rts1} (Figure S6A). We selected the 40 most affected sites, whose persistent phosphorylation until the end of the time course cannot be explained solely by delayed Cdk downregulation. These sites provide a resource for future studies into how PP2A^{Cdc55} and PP2A^{Rts1} promote mitotic exit (Figures S6B and S6C; Data S2 and S3, sheet 10). One of the phosphosites identified in cells lacking PP2A^{Cdc55} was Orc6 T146, confirming that PP2A^{Cdc55} contributes to Orc6 dephosphorylation (compare Figure 2B). Additional substrates implicated in DNA replication were also PP2A^{Cdc55} targets, including the DNA replication licensing factor Mcm4 and the large DNA polymerase α subunit Pol1. Substrates implicated in cytoskeletal organization included the actin regulators Abp1 and Pan1, as well as the septin Shs1 and formin Bni1. The identification of these substrates contributes to explaining the role of PP2A^{Cdc55} during mitotic exit.

31 of the 40 PP2A^{Cdc55} target sites were also contained in our PP2A^{Rts1} dataset. We therefore plotted the behavior of these phosphosites in control and both phosphatase deletion backgrounds (Figure S6D). This revealed that PP2A^{Rts1} is required together with PP2A^{Cdc55} for efficient dephosphorylation of all PP2A^{Cdc55} target sites.

We next explored the substrates most affected by the absence of PP2A^{Rts1}. These included the mitotic exit kinase Gin4, the mitotic exit network component Lte1, as well as the F-box protein Ufo1 (Figure S6C). All phosphosites affected by PP2A^{Rts1} were also subject to regulation by PP2A^{Cdc55} (Figure S6E). We reached the same conclusion from our replicate experiment (Figure S6F; Data S4). This suggests that PP2A^{Cdc55} and PP2A^{Rts1} have an overlapping substrate spectrum during mitotic exit, despite their distinct regulatory subunits.

Small linear interaction motifs are thought to provide phosphatase substrate specificity. PP2A^{Rts1} recognizes an LxxIxE motif ([LCVMIF][ST]P[ILVM]xE) (Hertz et al., 2016). 98 budding yeast proteins harbor at least one LxxIxE motif, of which 33 proteins were represented in our PP2A^{Rts1} dataset. In the control, phosphosites on only 10 of these proteins were dephosphorylated during mitotic exit. Their dephosphorylation depended on PP2A^{Rts1} (Figure S6G). This is consistent with a contribution of the LxxIxE motif for PP2A^{Rts1} recognition, but also highlights the difficulty with predicting functional small linear interaction motifs based on the amino acid sequence alone. Cdc14 recognizes a PxL motif (Kataria et al., 2018). Four dephosphorylated substrates in the Cdc14 dataset contained a PxL motif and these depended on Cdc14 for dephosphorylation. Their number was too small to allow a meaningful analysis of a possible contribution of the PxL motif to their dephosphorylation timing.

PP2A^{Cdc55} and PP2A^{Rts1} Sustain NDR and Aurora Kinase Motif Phosphorylation

While investigating phosphoproteome dynamics in cells lacking PP2A^{Rts1}, we noticed 264 phosphosites that are stable in control cells, but are dephosphorylated in the absence of PP2A^{Rts1} (Figure 6A). Therefore, PP2A^{Rts1} might be important to sustaining certain kinase(s), which maintain these phosphorylation events during mitotic exit. A sequence logo of the 264 affected sites revealed that an (R/K)x(S/T) Aurora kinase motif was enriched (Figure 6B). This suggests that PP2A^{Rts1} supports Aurora kinase

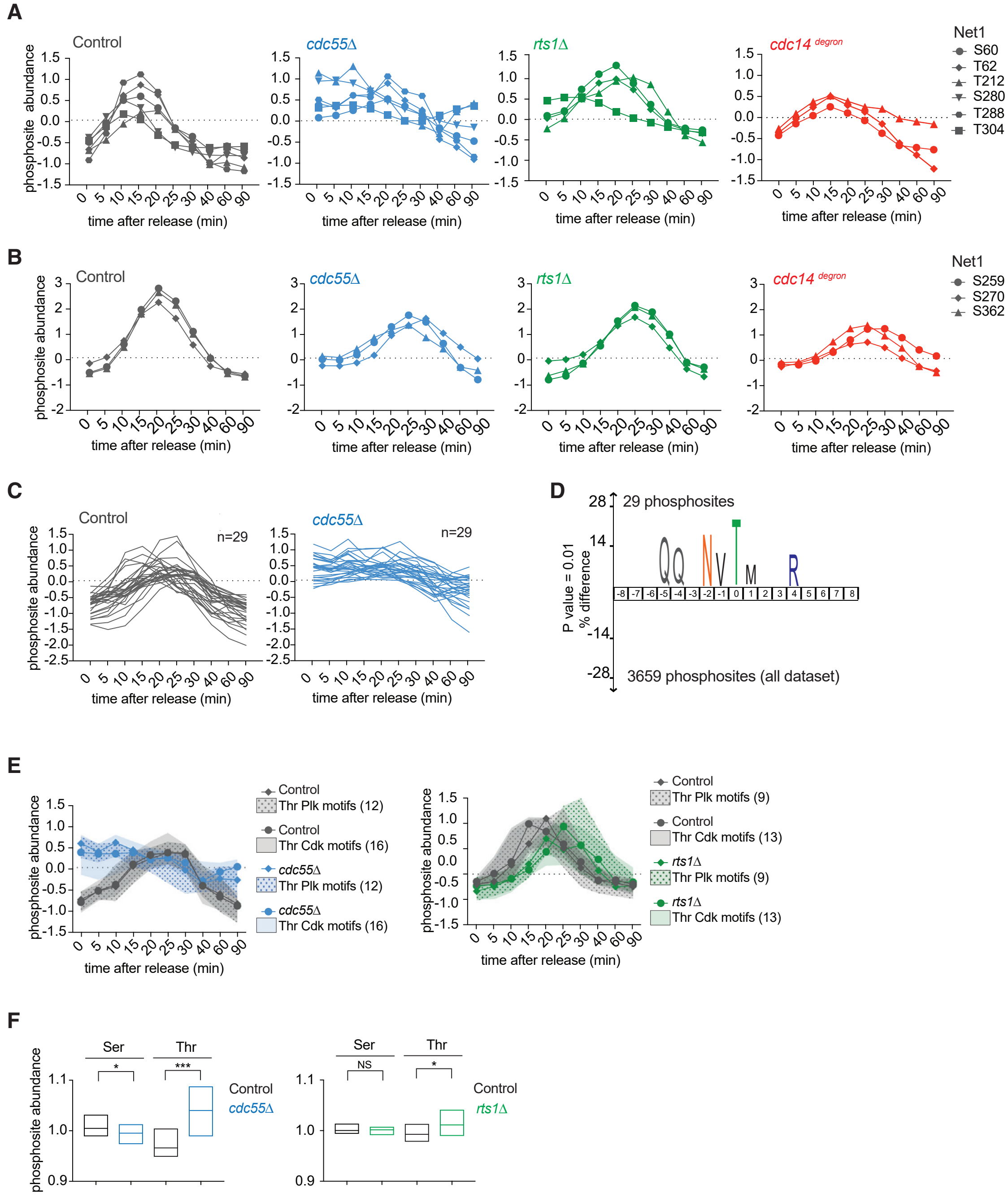

Figure 5. PP2A^{Cdc55} Shapes an Anaphase-Specific Phosphorylation Wave

(A) Profile plot of six Net1 phosphosites transiently phosphorylated in the control (black) and hyperphosphorylated in the *cdc55Δ* strain. The same phosphosites are also plotted in *rts1Δ* and *cdc14^{degron}* cells.

(B) Profile plot of three Net1 phosphosites transiently phosphorylated in the control (black) as well as in the *cdc55Δ* strain.

(C) 29 sites identified by correlation analysis in control and *cdc55Δ* cells.

(D) IceLogo motif analysis of the 29 phosphosites identified in (C). The phosphorylated residue is at position 0. Larger letter size indicates increasing enrichment; the threshold for enrichment detection was p = 0.01.

(E) Median intensity profile and interquartile range of transiently phosphorylated phosphosites that adhere to the two indicated kinase consensus motifs.

(F) Median phosphosite abundance over all 10 time points in control, *cdc55Δ*, and *rts1Δ* cells, grouped by phosphoacceptor amino acid. 2,948 and 3,491 serine sites and 711 and 880 threonine sites entered the analysis from the *cdc55Δ* and *rts1Δ* datasets, respectively. *cdc55Δ* *p = 0.038; **p = 0.0002. *rts1Δ* *p = 0.0103; NS, not significant, unpaired t test.

See also Figure S5 for design of the correlation analysis, full list of phosphosites identified, and global phosphosite abundance in the *cdc14^{degron}* dataset.

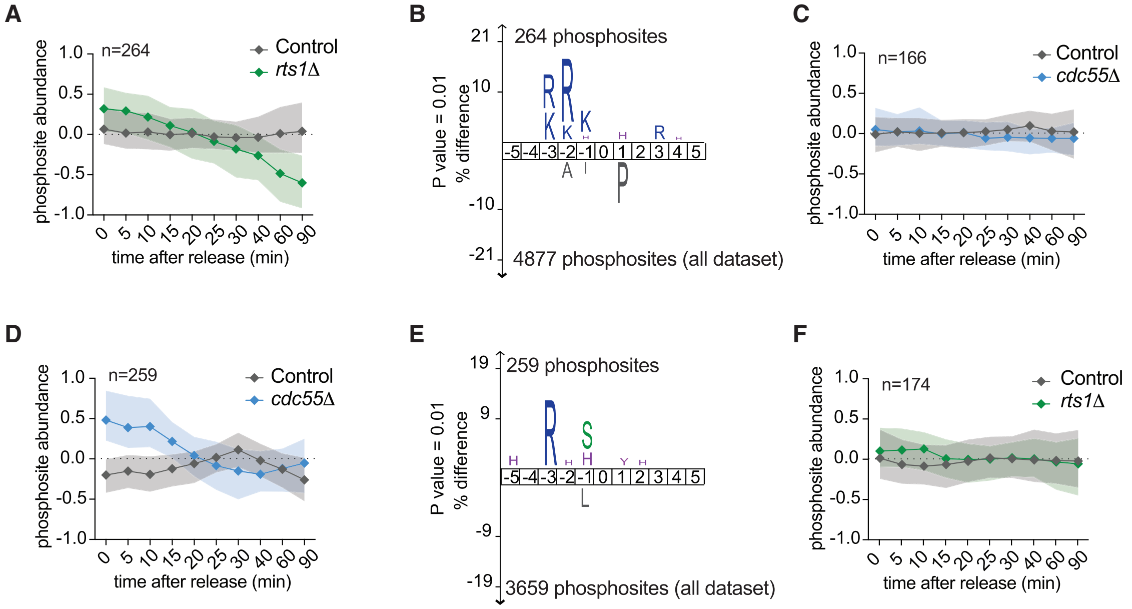

Figure 6. PP2A^{Cdc55} and PP2A^{Rts1} Sustain NDR and Aurora Kinase Motif Phosphorylation

(A) Median intensity profile and interquartile range of 264 phosphosites that are stable or phosphorylated in control, but dephosphorylated in *rts1Δ* cells.
(B) IceLogo motif analyses of these sites; the threshold for enrichment detection was p = 0.01.
(C) Median intensity profile and interquartile range of 166 phosphosites from (A) that are present in the *cdc55Δ* dataset.
(D) Median intensity profile and interquartile range of 259 phosphosites that are stable or phosphorylated in control, but dephosphorylated in *cdc55Δ* cells.
(E) IceLogo motif analyses of these sites; the threshold for enrichment detection was p = 0.01.
(F) Median intensity profile and interquartile range of 174 phosphosites from (D) that are present in the *rts1Δ* dataset.
See also Figure S6 for the identification of PP2A^{Cdc55} and PP2A^{Rts1} targets.

activity during mitotic exit. Consistent with this possibility, we found increased phosphorylation on two residues of the budding yeast Aurora kinase catalytic subunit Ipl1 in the absence of PP2A^{Rts1} (Figure S6H). The two other Aurora kinase complex subunits Bir1 and Sli15 were previously found to be hyperphosphorylated in cells lacking PP2A^{Rts1} (Zapata et al., 2014). Note that, in this case, the effect on Aurora kinase substrates was specific to PP2A^{Rts1}. 166 of the 264 affected phosphosites were also recorded in our PP2A^{Cdc55} dataset. The absence of PP2A^{Cdc55} had little impact on these sites (Figure 6C). This suggests that PP2A^{Rts1} plays a specific role in the regulation of Aurora kinase activity during mitotic exit.

We also found 259 phosphosites that are stable in control cells, but become dephosphorylated in the absence of PP2A^{Cdc55} (Figure 6D). Their sequence logo showed an enrichment in HxRxxS and RxxS NDR kinase motifs (Figure 6E). A subset of 174 of these sites were contained in our PP2A^{Rts1} dataset and these remained largely unaffected in the absence of PP2A^{Rts1} (Figure 6F). This suggests a specific role for PP2A^{Cdc55} in the regulation of NDR kinase phosphorylation during mitotic exit. Note that Cbk1 dephosphorylation, thought to promote its activation (Brace et al., 2011), is greatly compromised in the absence of PP2A^{Cdc55} (compare Figure 2B). These results demonstrate that PP2A^{Cdc55} and PP2A^{Rts1} not only make crucial contributions to protein dephosphorylation during mitotic exit, but additionally shape phosphoproteome dynamics by positively regulating late mitotic kinases.

Phosphatase Interplay Promotes Timely Mitotic Progression

While our phosphoproteome analysis revealed specific roles for each Cdc14, PP2A^{Cdc55}, and PP2A^{Rts1}, it also revealed a surprising degree of overlap. To investigate possible mechanisms for redundancy, we considered phosphoregulation of the phosphatases themselves and their regulators. The endosulfines Igo1/Igo2, as well as Zds1/Zds2, interact with and regulate PP2A^{Cdc55} (Juanes et al., 2013; Queralt and Uhlmann, 2008b). Igo2 phosphorylation was markedly reduced in cells lacking PP2A^{Cdc55}, as was a wave of Zds2 phosphorylation that we observed in control cells (Figures S7A and S7B). This suggests that feedback control operates between PP2A^{Cdc55} and its regulators. The Cdc14 phosphatase itself is also subject to phosphoregulation. In particular, Cdc14 S429 phosphorylation, which is thought to inhibit phosphatase activity (Li et al., 2014), was vastly reduced in the absence of PP2A^{Cdc55} (Figure 7A). S429 phosphorylation is high in control metaphase cells and declines during mitotic exit as Cdc14 becomes active. In cells lacking PP2A^{Cdc55}, S429 phosphorylation was low in metaphase and remained low throughout mitotic exit. Constitutively lower levels of Cdc14 phosphorylation were also apparent when observing Cdc14 electrophoretic mobility in cells lacking PP2A^{Cdc55} (Figure S7C). Premature Cdc14 release from the nucleolus in cells lacking PP2A^{Cdc55} (Queralt et al., 2006) might facilitate Cdc14 autodephosphorylation, thereby additionally compensating for the absence of PP2A^{Cdc55}. These considerations highlight that the

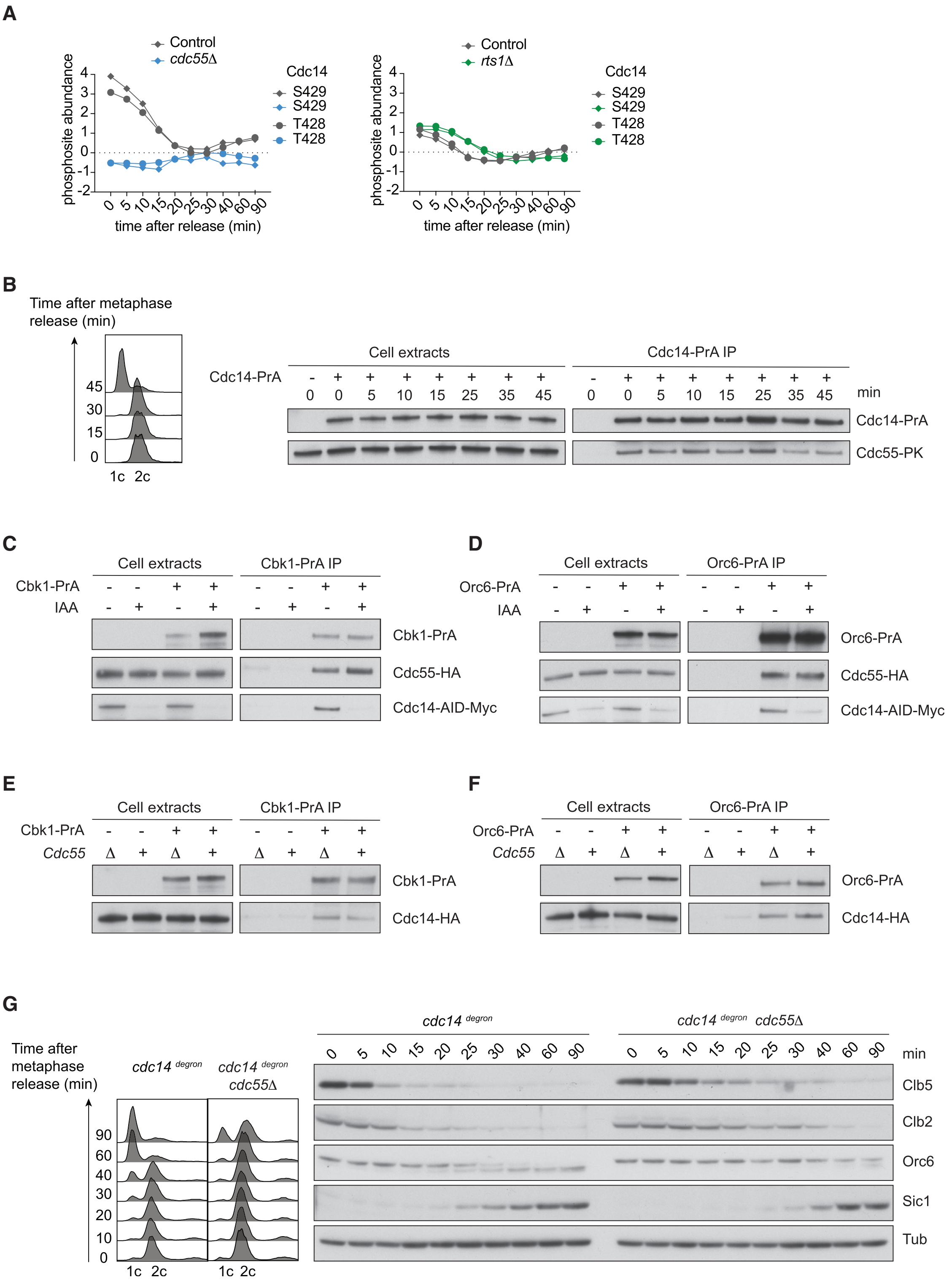

Figure 7. Phosphatase Interplay Promotes Timely Mitotic Progression

(A) Profile plot of Cdc14 phosphosites in control, *cdc55Δ*, and *rts1Δ* cells.

(B) A physical interaction between Cdc14 and PP2A^{Cdc55}. Cells were synchronized in metaphase and released. Protein A-tagged Cdc14 was precipitated from cell extracts at the indicated times and coprecipitation Cdc55 was analyzed by western blotting. Cell-cycle progression was monitored by FACS analysis of DNA content.

(legend continued on next page)

impact of phosphatases on each other, as well as on kinases, must be kept in mind when interpreting phosphorylation changes.

In addition to this interdependent regulation, we tested whether Cdc14 and PP2A^{Cdc55} engage in a direct physical interaction. We affinity purified protein A epitope tagged Cdc14 from budding yeast cell extracts and found that Cdc55 specifically coprecipitated with Cdc14, an interaction that was detectable throughout the course of mitotic exit (Figure 7B). This opens the possibility for direct cooperation between the two phosphatases.

Two examples where Cdc14 and PP2A^{Cdc55} cooperate to achieve complete substrate dephosphorylation are Orc6 and Cbk1 (compare Figures 2A and 2B). We therefore investigated whether Cdc14 and PP2A^{Cdc55} target these substrates in an interdependent manner. Affinity pulldowns of Cbk1 and Orc6 were used, which demonstrated a detectable interaction with both phosphatases. The interaction of Cbk1 and Orc6 with Cdc55 were unaffected by depletion of Cdc14 (Figures 7C and 7D). Likewise, Cbk1 and Orc6 interacted with Cdc14 in cells containing or lacking Cdc55 (Figures 7E and 7F). Thus, even though Cdc14 and PP2A^{Cdc55} coordinately target Cbk1 and Orc6 for dephosphorylation, they interact with their substrates independently of each other.

Finally, we directly investigated the joint contribution of Cdc14 and PP2A^{Cdc55} to mitotic progression. Cdc14 depletion and Cdc55 removal each caused a mitotic exit delay (compare Figures 1 and 2). When we depleted Cdc14 in a strain background lacking Cdc55, this caused a much longer mitotic exit delay. Cytokinesis happened very inefficiently and in only a subset of cells while Orc6 dephosphorylation was nearly absent (Figure 7G). Additive effects were also observed when Cdc14 was depleted from cells lacking Rts1 (Figure S7D). These results emphasize that in spite of overlapping roles, the phosphatases Cdc14, PP2A^{Cdc55}, and PP2A^{Rts1} make additive contributions to guarantee efficient substrate dephosphorylation during mitotic exit.

DISCUSSION

In this study, we employ time-resolved phosphoproteome analysis to reveal the contributions of three major phosphatases—Cdc14, PP2A^{Cdc55}, and PP2A^{Rts1}—to the protein phosphorylation and dephosphorylation program during budding yeast mitotic progression. Expanding upon the previous appreciation of Cdc14 as a key budding yeast mitotic exit phosphatase, we found that both PP2A^{Cdc55} and PP2A^{Rts1} make key contributions to phosphoproteome dynamics during mitotic progression and to successful completion of cell division.

Cdc14 Orders Mitotic Progression

Previous analyses of Cdc14 made use of temperature-sensitive alleles to inactivate the phosphatase (Visintin et al., 1998). Compromised Cdk downregulation in that case confounds the ability to identify direct Cdc14 targets. Here, we used partial conditional depletion of Cdc14. At the resulting depletion levels, Cdk downregulation occurred with only a small delay, allowing us to better define the substrate spectrum that is controlled by Cdc14. Note that residual Cdc14 activity means that this analysis will have underestimated the full contribution of Cdc14 to protein dephosphorylation.

Despite only partial depletion, we could identify numerous target proteins whose dephosphorylation patterns are affected by Cdc14. Most notably, we found a prominent impact of Cdc14 on sequential protein dephosphorylation during mitotic exit. We have in the past proposed a quantitative model showing how Cdc14 sequentially dephosphorylates its targets during mitotic exit (Bouchoux and Uhlmann, 2011). Early targets can be dephosphorylated by a small amount of Cdc14, even in the presence of persisting Cdk activity. Later targets require the Cdk-Cdc14 balance to shift farther toward Cdc14. Central to this model is not only decreasing Cdk activity during mitotic exit, but also increasing Cdc14 phosphatase activity. This is enacted in budding yeast by the gradual release of Cdc14 from Net1 inhibition in the nucleolus (Stegmeier and Amon, 2004). We imagine that an activity increase of a Cdk counteracting phosphatase is instrumental to control ordered substrate dephosphorylation. In vertebrates, this is likely PP2A in its antagonistic relationship with Cdk (Castro and Lorca, 2018). The role of Cdc14 in organisms other than budding yeast remains less clear. On the one hand, several species are known to be viable in the absence of Cdc14, including *C. elegans*. On the other hand, mitotic exit defects and cytokinesis failure have been reported following acute Cdc14 depletion from *C. elegans* embryos (Gruneberg et al., 2002; Saito et al., 2004). The contribution of three human Cdc14 isoforms CDC14A–C to mitosis and their possible redundancy with other phosphatases will be important to explore.

An Anaphase-Specific Phosphorylation Pattern Generated by PP2A^{Cdc55}

Even though our study has confirmed that Cdc14 makes an important contribution to budding yeast mitotic exit, we have also uncovered unanticipated roles for PP2A^{Cdc55} and PP2A^{Rts1}. We previously found that PP2A^{Cdc55} counteracts Cdk phosphorylation during interphase, in particular by dephosphorylating phosphothreonine residues (Godfrey et al., 2017). We now show that this role extends into mitosis, when

(C) Phosphatase-substrate interactions. Protein A-tagged Cbk1 was precipitated and the coprecipitation of PP2A^{Cdc55} was analyzed in extracts from metaphase arrested cells with or without Cdc14 depletion following auxin (IAA) addition.
(D) As in (C), but protein A-tagged Orc6 was precipitated.
(E) Protein A-tagged Cbk1 was precipitated and the coprecipitation Cdc14 was analyzed in extracts from metaphase-arrested control or *cdc55Δ* cells.
(F) As in (E), but protein A-tagged Orc6 was precipitated.
(G) Mitotic progression of control *cdc14^{degron}* and *cdc55Δ cdc14^{degron}* cells. Cell-cycle progression was monitored by FACS analysis of DNA content and western blotting against the indicated proteins.
See also Figure S7 for analysis of Igo2 and Zds2 phosphosites, Cdc14 phosphorylation by electrophoretic mobility shift, and mitotic exit kinetics of *rts1Δ cdc14^{degron}* cells.

$PP2A^{Cdc55}$ opposes phosphorylation by both Cdk and Plk kinases. A temporary dip in $PP2A^{Cdc55}$ activity, mediated by separase at anaphase onset, allows Cdk and Plk to gain the upper hand and instruct a transient anaphase-specific phosphorylation wave. How Cdk and Plk specificity of the anaphase wave is achieved is unclear. It could in part be due to the substrate specificity of PP2A, which is still incompletely understood. On the other hand, Cdk and Plk are two kinases that are at the peak of their activity during this time of the cell cycle. A decline of their counteracting phosphatase can therefore be expected to boost phosphorylation of their substrates. We do not currently know whether a similar anaphase-specific mode of substrate regulation operates in other organisms. Separase-dependent $PP2A^{Cdc55}$ downregulation is so far only known in budding yeast. However, sudden phosphorylation changes at the time of anaphase onset also occur in vertebrates (Mochida and Hunt, 2012). How such sudden phosphorylation changes are implemented in these organisms remains to be investigated.

Specificities and Cooperation between Mitotic Phosphatases

One of the great surprises of our study was the degree of cooperation between the three phosphatases Cdc14, $PP2A^{Cdc55}$, and $PP2A^{Rts1}$. Recent biochemical characterization of the three phosphatases has revealed distinct substrate targeting mechanisms. Cdc14 recognizes a PxL docking sequence and prefers to dephosphorylate phosphoserine within a pSPxK/R motif (Bremmer et al., 2012; Eissler et al., 2014; Kataria et al., 2018). In mammals, $PP2A^{B55}$ is attracted to a polybasic docking sequence and shows exquisite selectivity for threonine dephosphorylation, at least *in vitro* (Agostinis et al., 1990; Cundell et al., 2016). $PP2A^{B56}$ in turn recognizes LxxIxE sequences (Hertz et al., 2016; Nilsson, 2019; Wang et al., 2016). Based on this biochemical knowledge, one could have expected a strict division of labor between these phosphatases, in accordance with their substrate specificities. Instead, we found that many protein substrates, and indeed many individual phosphosites, are jointly controlled by two or more phosphatases.

Our phosphoproteome analysis revealed that $PP2A^{Cdc55}$ and $PP2A^{Rts1}$ share a majority of their substrates. We imagine that phosphatases make use of combinatorial substrate recognition to identify their targets. Affinity to either a regulatory subunit or the active site will contribute to substrate selection, thereby expanding the target range. An example of this is phosphothreonine dephosphorylation by Cdc14, usually disfavored by this phosphatase. Addition of a PxL substrate targeting sequence allows, albeit slow, phosphothreonine dephosphorylation (Kataria et al., 2018). In a similar way, a multitude of recognition modes might enable phosphatases to recognize their targets *in vivo*. If this is correct, then phosphatases might indeed show a broader substrate range when compared to kinases. The aggregate levels of phosphatase activity then become an important determinant of mitotic progression, with the three phosphatases Cdc14, $PP2A^{Cdc55}$, and $PP2A^{Rts1}$ all adding up to counteract kinases as cells progress through mitosis and return to G1.

STAR★METHODS

Detailed methods are provided in the online version of this paper and include the following:

- KEY RESOURCES TABLE
- LEAD CONTACT AND MATERIALS AVAIBILITY
- EXPERIMENTAL MODEL AND SUBJECT DETAILS
  - Strains
  - Culture
- METHOD DETAILS
  - Fluorescence Activated Cell Sorting Analysis of DNA Content
  - Immunofluorescence
  - Western Blotting
  - Affinity Coprecipitation
  - Cdk Kinase Assay
  - Extract Preparation for Phosphoproteome Analysis
  - Sample Preparation for Mass Spectrometry
- QUANTIFICATION AND STATISTICAL ANALYSIS
  - Mass Spectrometry Data Analysis
  - Phosphosite Abundance Change Analysis
  - Clustering and Correlation Analysis
  - Icelogo Sequence Analysis
- DATA AND CODE AVAILABILITY

SUPPLEMENTAL INFORMATION

Supplemental Information can be found online at https://doi.org/10.1016/j.celrep.2019.10.041.

ACKNOWLEDGMENTS

We would like to thank K. Shirahige for the Scc1 antibody, M. Godfrey, M. Kataria, K. Wassmann, and all our laboratory members for insightful discussions and critical reading of the manuscript. This work was supported by The Francis Crick Institute, which receives its core funding from Cancer Research UK (FC001198), the UK Medical Research Council (FC001198), and the Wellcome Trust (FC001198).

AUTHOR CONTRIBUTIONS

S.A.T. and F.U. conceived the study, S.A.T. performed most of the experiments and data analyses, L.H. contributed the $PP2A^{Rts1}$ experiments, A.W.J. and A.P.S. performed the mass spectrometry proteomics analysis, G.K. supported the data analysis, and S.A.T. and F.U. wrote the manuscript with input from all coauthors.

DECLARATION OF INTERESTS

The authors declare no competing interests.

Received: March 11, 2019
Revised: September 27, 2019
Accepted: October 10, 2019
Published: November 12, 2019

REFERENCES

Afonso, O., Figueiredo, A.C., and Maiato, H. (2017). Late mitotic functions of Aurora kinases. Chromosoma *126*, 93–103.

Agostinis, P., Goris, J., Pinna, L.A., Marchiori, F., Perich, J.W., Meyer, H.E., and Merlevede, W. (1990). Synthetic peptides as model substrates for the

study of the specificity of the polycation-stimulated protein phosphatases. Eur. J. Biochem. *189*, 235–241.

Azzam, R., Chen, S.L., Shou, W., Mah, A.S., Alexandru, G., Nasmyth, K., Annan, R.S., Carr, S.A., and Deshaies, R.J. (2004). Phosphorylation by cyclin B-Cdk underlies release of mitotic exit activator Cdc14 from the nucleolus. Science *305*, 516–519.

Baro, B., Játiva, S., Calabria, I., Vinaixa, J., Bech-Serra, J.J., de LaTorre, C., Rodrigues, J., Hernáez, M.L., Gil, C., Barceló-Batllori, S., et al. (2018). SILAC-based phosphoproteomics reveals new PP2A-Cdc55-regulated processes in budding yeast. Gigascience *7*, giy047.

Bloom, J., and Cross, F.R. (2007). Novel role for Cdc14 sequestration: Cdc14 dephosphorylates factors that promote DNA replication. Mol. Cell. Biol. *27*, 842–853.

Botchkarev, V.V., Jr., and Haber, J.E. (2018). Functions and regulation of the Polo-like kinase Cdc5 in the absence and presence of DNA damage. Curr. Genet. *64*, 87–96.

Bouchoux, C., and Uhlmann, F. (2011). A quantitative model for ordered Cdk substrate dephosphorylation during mitotic exit. Cell *147*, 803–814.

Brace, J., Hsu, J., and Weiss, E.L. (2011). Mitotic exit control of the Saccharomyces cerevisiae Ndr/LATS kinase Cbk1 regulates daughter cell separation after cytokinesis. Mol. Cell. Biol. *31*, 721–735.

Breitkreutz, A., Choi, H., Sharom, J.R., Boucher, L., Neduva, V., Larsen, B., Lin, Z.Y., Breitkreutz, B.J., Stark, C., Liu, G., et al. (2010). A global protein kinase and phosphatase interaction network in yeast. Science *328*, 1043–1046.

Bremmer, S.C., Hall, H., Martinez, J.S., Eissler, C.L., Hinrichsen, T.H., Rossie, S., Parker, L.L., Hall, M.C., and Charbonneau, H. (2012). Cdc14 phosphatases preferentially dephosphorylate a subset of cyclin-dependent kinase (Cdk) sites containing phosphoserine. J. Biol. Chem. *287*, 1662–1669.

Castro, A., and Lorca, T. (2018). Greatwall kinase at a glance. J. Cell Sci. *131*, jcs222364.

Chen, M.J., Dixon, J.E., and Manning, G. (2017). Genomics and evolution of protein phosphatases. Sci. Signal. *10*, eaag1796.

Colaert, N., Helsens, K., Martens, L., Vandekerckhove, J., and Gevaert, K. (2009). Improved visualization of protein consensus sequences by iceLogo. Nat. Methods *6*, 786–787.

Cundell, M.J., Hutter, L.H., Nunes Bastos, R., Poser, E., Holder, J., Mohammed, S., Novák, B., and Barr, F.A. (2016). A PP2A-B55 recognition signal controls substrate dephosphorylation kinetics during mitotic exit. J. Cell Biol. *214*, 539–554.

Dobbelaere, J., Gentry, M.S., Hallberg, R.L., and Barral, Y. (2003). Phosphorylation-dependent regulation of septin dynamics during the cell cycle. Dev. Cell *4*, 345–357.

Eissler, C.L., Mazón, G., Powers, B.L., Savinov, S.N., Symington, L.S., and Hall, M.C. (2014). The Cdk/cDc14 module controls activation of the Yen1 holliday junction resolvase to promote genome stability. Mol. Cell *54*, 80–93.

Foiani, M., Marini, F., Gamba, D., Lucchini, G., and Plevani, P. (1994). The B subunit of the DNA polymerase alpha-primase complex in Saccharomyces cerevisiae executes an essential function at the initial stage of DNA replication. Mol. Cell. Biol. *14*, 923–933.

Gelens, L., Qian, J., Bollen, M., and Saurin, A.T. (2018). The Importance of Kinase-Phosphatase Integration: Lessons from Mitosis. Trends Cell Biol. *28*, 6–21.

Godfrey, M., Touati, S.A., Kataria, M., Jones, A., Snijders, A.P., and Uhlmann, F. (2017). PP2A^{Cdc55} Phosphatase Imposes Ordered Cell-Cycle Phosphorylation by Opposing Threonine Phosphorylation. Mol. Cell *65*, 393–402.e3.

Gruneberg, U., Glotzer, M., Gartner, A., and Nigg, E.A. (2002). The CeCDC-14 phosphatase is required for cytokinesis in the Caenorhabditis elegans embryo. J. Cell Biol. *158*, 901–914.

Harvey, S.L., Enciso, G., Dephoure, N., Gygi, S.P., Gunawardena, J., and Kellogg, D.R. (2011). A phosphatase threshold sets the level of Cdk1 activity in early mitosis in budding yeast. Mol. Biol. Cell *22*, 3595–3608.

Hein, J.B., Hertz, E.P.T., Garvanska, D.H., Kruse, T., and Nilsson, J. (2017). Distinct kinetics of serine and threonine dephosphorylation are essential for mitosis. Nat. Cell Biol. *19*, 1433–1440.

Hertz, E.P.T., Kruse, T., Davey, N.E., López-Méndez, B., Sigurðsson, J.O., Montoya, G., Olsen, J.V., and Nilsson, J. (2016). A Conserved Motif Provides Binding Specificity to the PP2A-B56 Phosphatase. Mol. Cell *63*, 686–695.

Imtiaz, A., Belyantseva, I.A., Beirl, A.J., Fenollar-Ferrer, C., Bashir, R., Bukhari, I., Bouzid, A., Shaukat, U., Azaiez, H., Booth, K.T., et al. (2018). CDC14A phosphatase is essential for hearing and male fertility in mouse and human. Hum. Mol. Genet. *27*, 780–798.

Jaspersen, S.L., and Morgan, D.O. (2000). Cdc14 activates cdc15 to promote mitotic exit in budding yeast. Curr. Biol. *10*, 615–618.

Játiva, S., Calabria, I., Moyano-Rodriguez, Y., Garcia, P., and Queralt, E. (2019). Cdc14 activation requires coordinated Cdk1-dependent phosphorylation of Net1 and PP2A-Cdc55 at anaphase onset. Cell. Mol. Life Sci. *76*, 3601–3620.

Jin, F., Liu, H., Liang, F., Rizkallah, R., Hurt, M.M., and Wang, Y. (2008). Temporal control of the dephosphorylation of Cdk substrates by mitotic exit pathways in budding yeast. Proc. Natl. Acad. Sci. USA *105*, 16177–16182.

Juanes, M.A., Khoueiry, R., Kupka, T., Castro, A., Mudrak, I., Ogris, E., Lorca, T., and Piatti, S. (2013). Budding yeast greatwall and endosulfines control activity and spatial regulation of PP2A(Cdc55) for timely mitotic progression. PLoS Genet. *9*, e1003575.

Kao, L., Wang, Y.T., Chen, Y.C., Tseng, S.F., Jhang, J.C., Chen, Y.J., and Teng, S.C. (2014). Global analysis of cdc14 dephosphorylation sites reveals essential regulatory role in mitosis and cytokinesis. Mol. Cell. Proteomics *13*, 594–605.

Kataria, M., Mouilleron, S., Seo, M.H., Corbi-Verge, C., Kim, P.M., and Uhlmann, F. (2018). A PxL motif promotes timely cell cycle substrate dephosphorylation by the Cdc14 phosphatase. Nat. Struct. Mol. Biol. *25*, 1093–1102.

Kuilman, T., Maiolica, A., Godfrey, M., Scheidel, N., Aebersold, R., and Uhlmann, F. (2015). Identification of Cdk targets that control cytokinesis. EMBO J. *34*, 81–96.

Li, Y., Cross, F.R., and Chait, B.T. (2014). Method for identifying phosphorylated substrates of specific cyclin/cyclin-dependent kinase complexes. Proc. Natl. Acad. Sci. USA *111*, 11323–11328.

Lianga, N., Williams, E.C., Kennedy, E.K., Doré, C., Pilon, S., Girard, S.L., Deneault, J.S., and Rudner, A.D. (2013). A Wee1 checkpoint inhibits anaphase onset. J. Cell Biol. *201*, 843–862.

Manchado, E., Guillamot, M., de Cárcer, G., Eguren, M., Trickey, M., García-Higuera, I., Moreno, S., Yamano, H., Cañamero, M., and Malumbres, M. (2010). Targeting mitotic exit leads to tumor regression in vivo: Modulation by Cdk1, Mastl, and the PP2A/B55α,δ phosphatase. Cancer Cell *18*, 641–654.

Meitinger, F., Boehm, M.E., Hofmann, A., Hub, B., Zentgraf, H., Lehmann, W.D., and Pereira, G. (2011). Phosphorylation-dependent regulation of the F-BAR protein Hof1 during cytokinesis. Genes Dev. *25*, 875–888.

Minshull, J., Straight, A., Rudner, A.D., Dernburg, A.F., Belmont, A., and Murray, A.W. (1996). Protein phosphatase 2A regulates MPF activity and sister chromatid cohesion in budding yeast. Curr. Biol. *6*, 1609–1620.

Mochida, S., and Hunt, T. (2012). Protein phosphatases and their regulation in the control of mitosis. EMBO Rep. *13*, 197–203.

Mochida, S., Ikeo, S., Gannon, J., and Hunt, T. (2009). Regulated activity of PP2A-B55 δ is crucial for controlling entry into and exit from mitosis in Xenopus egg extracts. EMBO J. *28*, 2777–2785.

Morgan, D.O. (2007). The Cell Cycle (New Science Press).

Neitzel, L.R., Broadus, M.R., Zhang, N., Sawyer, L., Wallace, H.A., Merkle, J.A., Jodoin, J.N., Sitaram, P., Crispi, E.E., Rork, W., et al. (2018). Characterization of a cdc14 null allele in Drosophila melanogaster. Biol. Open *7*, bio035394.

Nilsson, J. (2019). Protein phosphatases in the regulation of mitosis. J. Cell Biol. *218*, 395–409.

Nishimura, K., Fukagawa, T., Takisawa, H., Kakimoto, T., and Kanemaki, M. (2009). An auxin-based degron system for the rapid depletion of proteins in nonplant cells. Nat. Methods *6*, 917–922.

O'Reilly, N., Charbin, A., Lopez-Serra, L., and Uhlmann, F. (2012). Facile synthesis of budding yeast a-factor and its use to synchronize cells of α mating type. Yeast *29*, 233–240.

Peplowska, K., Wallek, A.U., and Storchova, Z. (2014). Sgo1 regulates both condensin and Ipl1/Aurora B to promote chromosome biorientation. PLoS Genet. *10*, e1004411.

Powers, B.L., and Hall, M.C. (2017). Re-examining the role of Cdc14 phosphatase in reversal of Cdk phosphorylation during mitotic exit. J. Cell Sci. *130*, 2673–2681.

Queralt, E., and Uhlmann, F. (2008a). Cdk-counteracting phosphatases unlock mitotic exit. Curr. Opin. Cell Biol. *20*, 661–668.

Queralt, E., and Uhlmann, F. (2008b). Separase cooperates with Zds1 and Zds2 to activate Cdc14 phosphatase in early anaphase. J. Cell Biol. *182*, 873–883.

Queralt, E., Lehane, C., Novák, B., and Uhlmann, F. (2006). Downregulation of PP2A(Cdc55) phosphatase by separase initiates mitotic exit in budding yeast. Cell *125*, 719–732.

Saito, R.M., Perreault, A., Peach, B., Satterlee, J.S., and van den Heuvel, S. (2004). The CDC-14 phosphatase controls developmental cell-cycle arrest in C. elegans. Nat. Cell Biol. *6*, 777–783.

Schmitz, M.H.A., Held, M., Janssens, V., Hutchins, J.R.A., Hudecz, O., Ivanova, E., Goris, J., Trinkle-Mulcahy, L., Lamond, A.I., Poser, I., et al. (2010). Live-cell imaging RNAi screen identifies PP2A-B55α and importin-β1 as key mitotic exit regulators in human cells. Nat. Cell Biol. *12*, 886–893.

Stegmeier, F., and Amon, A. (2004). Closing mitosis: the functions of the Cdc14 phosphatase and its regulation. Annu. Rev. Genet. *38*, 203–232.

Tamborrini, D., Juanes, M.A., Ibanes, S., Rancati, G., and Piatti, S. (2018). Recruitment of the mitotic exit network to yeast centrosomes couples septin displacement to actomyosin constriction. Nat. Commun. *9*, 4308.

Tomson, B.N., Rahal, R., Reiser, V., Monje-Casas, F., Mekhail, K., Moazed, D., and Amon, A. (2009). Regulation of Spo12 phosphorylation and its essential role in the FEAR network. Curr. Biol. *19*, 449–460.

Touati, S.A., Kataria, M., Jones, A.W., Snijders, A.P., and Uhlmann, F. (2018). Phosphoproteome dynamics during mitotic exit in budding yeast. EMBO J. *37*, e98745.

Uhlmann, F., Lottspeich, F., and Nasmyth, K. (1999). Sister-chromatid separation at anaphase onset is promoted by cleavage of the cohesin subunit Scc1. Nature *400*, 37–42.

Uhlmann, F., Bouchoux, C., and López-Avilés, S. (2011). A quantitative model for cyclin-dependent kinase control of the cell cycle: revisited. Philos. Trans. R. Soc. Lond. B Biol. Sci. *366*, 3572–3583.

Visintin, R., Craig, K., Hwang, E.S., Prinz, S., Tyers, M., and Amon, A. (1998). The phosphatase Cdc14 triggers mitotic exit by reversal of Cdk-dependent phosphorylation. Mol. Cell *2*, 709–718.

Wang, J., Wang, Z., Yu, T., Yang, H., Virshup, D.M., Kops, G.J.P.L., Lee, S.H., Zhou, W., Li, X., Xu, W., and Rao, Z. (2016). Crystal structure of a PP2A B56-BubR1 complex and its implications for PP2A substrate recruitment and localization. Protein Cell *7*, 516–526.

Wang, Y., and Burke, D.J. (1997). Cdc55p, the B-type regulatory subunit of protein phosphatase 2A, has multiple functions in mitosis and is required for the kinetochore/spindle checkpoint in Saccharomyces cerevisiae. Mol. Cell. Biol. *17*, 620–626.

Weiss, E.L. (2012). Mitotic exit and separation of mother and daughter cells. Genetics *192*, 1165–1202.

Zapata, J., Dephoure, N., Macdonough, T., Yu, Y., Parnell, E.J., Mooring, M., Gygi, S.P., Stillman, D.J., and Kellogg, D.R. (2014). PP2ARts1 is a master regulator of pathways that control cell size. J. Cell Biol. *204*, 359–376.

STAR★METHODS

KEY RESOURCES TABLE

REAGENT or RESOURCE	SOURCE	IDENTIFIER
Antibodies		
Mouse monoclonal anti-Scc1 (362 D11B10) (budding yeast)	Gift from Shirahige Lab	N/A
Mouse monoclonal anti- α-tubulin	Cell Services Science Technology Platform, The Francis Crick Institute	N/A
Mouse monoclonal anti-V5	Bio-Rad	Cat# MCA1360; RRID:AB_322378
Mouse monoclonal anti-HA (F7)	Santa Cruz	Cat# sc-7392; RRID:AB_627809
Mouse monoclonal anti-HA (12CA5)	Cell Services, The Francis Crick Institute	N/A
Goat monoclonal anti-c-Myc	Bethyl	A190-204A; RRID:AB_66865
Rabbit monoclonal anti-Clb2	Santa Cruz	Discontinued
Rabbit monoclonal anti-Clb5	Santa Cruz	Discontinued
Rabbit monoclonal anti-Sic1	Santa Cruz	Discontinued
Mouse monoclonal anti-Orc6	Cell Services, The Francis Crick Institute	N/A
Rat monoclonal anti- α-tubulin (YOL1/3)	Bio-Rad	Cat# MCA78G; RRID:AB_325005
Anti-mouse IgG CY3	Jackson Immunoresearch	Cat# 115-166-003; RRID:AB_2338699
Anti-rat IgG Alexa Fluor 594	Molecular Probes	
Rabbit Peroxidase Anti-Peroxidase	Sigma-Aldrich	Cat# P1291; RRID:AB_1079562
Anti-mouse IgG (HRP-conjugated)	GE Healthcare	Cat# NA931; RRID:AB_772210
Anti-rabbit IgG (HRP-conjugated)	GE Healthcare	Cat# NA934; RRID:AB_772206
Anti-goat IgG (HRP-conjugated)	Abcam	Cat# ab97110; RRID:AB_10679463
Chemicals, Peptides, and Recombinant Proteins		
α-factor	Peptide Chemistry Science Technology Platform, The Francis Crick institute	N/A
Nocodazole	Sigma-Aldrich	Cat# M1404
Indole-3-acetic acid (IAA)	Sigma-Aldrich	Cat# I3750
G418	Sigma-Aldrich	Cat# G8618
Formaldehyde solution	Sigma-Aldrich	Cat# 252549
cOmplete EDTA-Free Protease Inhibitor Cocktail	Sigma-Aldrich	Cat# 04693132001
Zymoliase 100T	MP	Cat# 320931
Benzonase® Nuclease	Sigma-Aldrich	Cat# E1014
RNase A	Sigma-Aldrich	Cat# 10109169001
Protein Assay Dye	Bio-Rad	Cat# 5000006
Propidium iodide solution	Sigma-Aldrich	Cat# P4864
GelRed® Nucleic Acid Gel Stain	Biotium	Cat# 41003-1
ATP	Roche	Cat# 11140965001
ATP^{33P}	Hartmann	SCF301
Histone H1	Roche	Discontinued
Pierce Trypsin Protease, MS Grade	ThermoFisher	Cat#90058
Critical Commercial Assays		
InFusion HD cloning kit	Clontech Laboratories	Cat# 639634
CloneAmp HiFi PCR Premix	Clontech Laboratories	Cat# 639298
Dynabeads Protein A	ThermoFisher	Cat# 10002D
Dynabeads M-270 Epoxy	ThermoFisher	Cat# 14302D
Rabbit immunoglobulin G (IgG)	Sigma-Aldrich	Cat# I5006

(*Continued on next page*)

LEAD CONTACT AND MATERIALS AVAIBILITY

Continued

REAGENT or RESOURCE	SOURCE	IDENTIFIER
ECL Prime Western Blotting Detection Regent	GE Healthcare	Cat# RPN2232
TMT 10plex Isobaric Label Reagent Set 1 × 0.8 mg	ThermoFisher	Cat#90110
Pierce TiO2 Phosphopeptide Enrichment Spin Kits	ThermoFisher	Cat#88303
High-Select Fe-NTA Phosphopeptide Enrichment Kit	ThermoFisher	Cat#A32992
UltiMate 3000 HPLC System	ThermoFisher	Cat#5041.0010
EASY-Spray C18 column, 75 μm × 50 cm	ThermoFisher	Cat#ES803
Orbitrap Fusion Lumos Tribrid Mass Spectrometer	ThermoFisher	Cat#IQLAAEGAAPFADBMBCX
**Deposited Data**		
The full mass spectrometry proteomics data obtained in this study have been deposited with the ProteomeXchange Consortium via the PRIDE partner repository	this paper	PXD012860
Unprocessed gel images presented in this manuscript can be found on Mendeley	this paper	https://doi.org/10.17632/tspgywx7g3.1
**Experimental Models: Organisms/Strains**		
All *Saccharomyces cerevisiae* strains used in this study are listed in Data S5.	Lab stock and this study	N/A
Escherichia coli DH5α competent cells	New England Biolabs	Cat# C2987U
**Software and Algorithms**		
Snapgene v2.6	GSL Biotech	https://www.snapgene.com
FlowJo v10.1	FlowJo	https://www.flowjo.com
Prism v7.0c	GraphPad	https://www.graphpad.com/scientific-software/prism/
ImageJ v1.50c	ImageJ	https://imagej.nih.gov/ij/
Perseus v1.4.0.2	Perseus	https://maxquant.net/perseus/
MaxQuant v1.5.0.13	MaxQuant	https://www.maxquant.org
Icelogo	Colaert et al., 2009	https://iomics.ugent.be/icelogoserver/

Epitope tagging of endogenous genes and gene deletion were performed using polymerase chain reaction (PCR)-based gene [illegible]

25°C. α-factor (7.5 μg/ml) was used for cell synchronization in G1 as described (O'Reilly et al., 2012). Nocodazole (6 μg/ml) [illegible]

[illegible] and the datafiles were curated using FlowJo software (FlowJo LLC).

Immunofluorescence

Aliquots of the cultures were taken at the indicated time intervals and fixed overnight in cold fixation buffer (100 mM potassium phosphate pH 6.4, 0.5 mM MgCl2, 3.7% formaldehyde). In brief, cells were spheroplasted in buffer containing 28 mM β-mercaptoethanol and 20 U/μl Zymolase T-100 by incubation at 37°C for 45 minutes. Immunofluorescence staining against tubulin was performed to evaluate spindle elongation during anaphase and spindle disassembly during mitotic exit. Cells were counterstained with the DNA binding dye Hoechst to evaluate DNA segregation. Fluorescent images were acquired using an Axioplan 2 Imaging microscope (Zeiss) equipped with a 100x (NA = 1.45) Plan-Neofluar objective and an ORCA-ER camera (Hamamatsu). Antibodies used for immunofluorescence are listed in the Key Resource Table.

Western Blotting

Protein extracts for western blotting were prepared following cell fixation using trichloroacetic acid (Foiani et al., 1994). 1 mL culture (OD_{600} = 0.3) was resuspended in 1ml of 20% trichloracetic acid and kept an hour on ice before being washed in 1 mL of 1M Tris-Base. Pellet were resuspended in 100 μL of 2X Laemmli buffer, boiled for 5min and clarified by centrifugation. Bio-Rad protein assay was used to evaluate protein concentration and 15 μg of proteins were separated by SDS–polyacrylamide gel electrophoresis before being transferred to nitrocellulose membranes. Antibodies used for Western detection are listed in the Key Resource Table.

Affinity Coprecipitation

Cell extracts were prepared in lysis buffer (50 mM HEPES-KOH pH 7.9, 100 mM NaCl, 2.5 mM $MgCl_2$, 10% glycerol, 0.25% Triton X-100, 0.5 mM TCEP, protease, phosphatase inhibitors and benzonase) using glass beads breakage in a refrigerated Multi-Beads Shocker. Extracts were cleared by centrifugation, then further cleared with Dynabeads Protein A (ThermoFisher) and incubated with Dynabeads IgG to retrieve protein A-tagged proteins of interest. Following washes, elution was carried out in SDS-PAGE loading buffer.

Cdk Kinase Assay

Cell extracts were prepared in lysis buffer (50 mM HEPES-KOH pH 7.9, 100 mM NaCl, 2.5 mM $MgCl_2$, 10% glycerol, 0.25% Triton X-100, 0.5 mM TCEP, protease, phosphatase inhibitors and benzonase) using glass beads breakage in a refrigerated Multi-Beads Shocker. Extracts were cleared by centrifugation and incubated with Dynabeads Protein A, previously ligated to an HA-specific antibody to pull down HA epitope-tagged Cdc28. Beads were extensively washed in lysis buffer and equilibrated in kinase buffer (50 mM HEPES-KOH pH 7.9, 150 mM NaCl, 10 mM $MgCl_2$, 0.2% Triton X-100 and phosphatase inhibitors) before performing the kinase reaction. Histone H1 phosphorylation reactions were carried out in kinase buffer containing 15 μM of histone H1 and 660 μM ATP, including 5.5 nM γ-^{33}P-ATP, for 15 minutes at 30°C. Reactions were terminated by addition of SDS-PAGE loading buffer and resolved by 12% SDS-PAGE. The gel was fixed, dried and exposed to a Phosphorimager screen (GE Healthcare).

Extract Preparation for Phosphoproteome Analysis

Control and mutant cells were grown overnight in rich YP medium supplemented with 2% raffinose + 2% galactose (YP Raff + Gal) at 25°C, arrested in mitosis by filtration and release into medium containing raffinose only. Following arrest, an aliquot of the cultures was harvested before readdition of galactose. Nine further aliquots were taken at the indicated time points. Cells were collected by centrifugation and resuspended in 100% (w/v) trichloroacetic acid solution for protein fixation. Following acetone washes, cells were resuspended in lysis buffer (50 mM ammonium bicarbonate, 5 mM EDTA pH 7.5, 8 M urea) and opened by glass bead breakage. Protein extracts were cleared by centrifugation.

Sample Preparation for Mass Spectrometry

300 μg of each protein sample was reduced with 5 mM dithiothreitol (DTT) for 25 minutes at 56°C, alkylated with 10 mM iodoacetamide for 30 minutes at room temperature in the dark, then quenched with 7.5 M DTT. Samples were diluted with 50 mM HEPES pH 8.5 to reduce the urea concentration to < 2 M, prior to trypsin digestion overnight at 37°C. Peptides were then acidified and desalted using a C18 SepPak column under vacuum and dried. The samples were arranged in sets of ten and labeled using the TMT10plex Isobaric Label Reagent Set (ThermoFisher), as per the manufacturer's instructions. Following labeling and mixing, multiplexed samples were again desalted using a C18 SepPak column. Phosphopeptide enrichment was performed by Sequential Enrichment from Metal Oxide Affinity Chromatography (SMOAC, ThermoFisher) with initial enrichment using the High-Select TiO_2 Phosphopeptide Enrichment Kit followed by the High-Select Fe-NTA Phosphopeptide Enrichment Kit (both Thermo Scientific) for the non-bound flowthrough fractions. Phosphopeptides were eluted, desalted using a C18 StageTip and analyzed in triplicate on an Orbitrap Fusion Lumos mass spectrometer (Thermo Fisher) coupled to an UltiMate 3000 HPLC system for on-line liquid chromatographic separation. Each run consisted of a three hour gradient elution from a 75 μm × 50 cm C18 column.

QUANTIFICATION AND STATISTICAL ANALYSIS

Mass Spectrometry Data Analysis

MaxQuant (version 1.5.0.13) was used for all data processing. The data was searched against a UniProt extracted *Saccharomyces cerevisiae* proteome FASTA file, amended to include common contaminants. Default MaxQuant parameters were used with the following adjustments. Phospho(STY) was added as a variable modification, MaxQuant output files were imported into Perseus (version 1.4.0.2). All reporter intensities were log_2 transformed and only phosphosites which were quantified in all 10 channels in at least one TMT group were retained. Reporter intensities were normalized by subtracting the median value for a specific TMT channel and then by subtracting the median value of each phosphosite across the time course. 61%, 68% and 72% of the phosphosites were quantified in all 10 time points of the Cdc14, $PP2A^{Cdc55}$ and $PP2A^{Rts1}$ experimental datasets, respectively. Missing values were imputed by calculating the mean of the two adjacent time points. Smoothing was then performed by replacing each technical or imputed value by the mean of the two adjacent time points. For both imputation and smoothing of the 0 and 90 minute time points, only the 5 and 60 minute values, respectively, were used. For the analyses shown in Figures 3 and S4, phosphosite intensities were log transformed and normalized to 0 at time point zero. For Figure 4, phosphosite intensities are in linear scale and normalized to 1. Figures 5, 6, S5, S6, S7, and S8 show phosphosite intensities on a log scale.

Phosphosite Abundance Change Analysis

Phosphosite abundances at time 0 were normalized. A 1.5 fold decrease in phosphosite abundance, or a 1.5 fold increase, over at least two consecutive time points was required to meet classification as dephosphorylated or phosphorylated, respectively. Phosphosites that showed a less than 1.5 fold change were considered stable. In Figures 4 and S4, the time point of dephosphorylation or phosphorylation was then assigned as the time when the threshold was first passed.

Clustering and Correlation Analysis

Correlation analyses in Figures 5 and S6 were performed in Perseus after designing expected profiles. Phosphosites with the lowest distance from the expected profiles were selected. For the hierarchical clustering analysis in Figures S3D–S3F and S6F, In-transformed datasets were normalized to 0 in metaphase (0 minutes) in the control. They were then subjected to hierarchical clustering with complete agglomeration of an Euclidean metric, to preserve differences in absolute phosphorylation levels. The dendrogram

was cut into 12 subtrees throughout, an arbitrary number thought to be at least as large as the number of different behavior patterns.

Icelogo Sequence Analysis

Amino acid distributions surrounding phosphosites was analyzed using IceLogo (Colaert et al., 2009). Phosphoresidues are placed at the central position within the sequence logo. A p-value = 0.01 was applied as detection threshold when using IceLogo. Percentage difference reflects the frequency of an amino acid in the indicated category, at a given position, compared to its frequency in the whole dataset, from where the category had been extracted.

DATA AND CODE AVAILABILITY

The full mass spectrometry proteomics data obtained in this study have been deposited with the ProteomeXchange Consortium via the PRIDE partner repository with the dataset identifier PXD012860.

Cell Reports, Volume *29*

Supplemental Information

Cdc14 and PP2A Phosphatases Cooperate to Shape Phosphoproteome Dynamics during Mitotic Exit

Sandra A. Touati, Lorena Hofbauer, Andrew W. Jones, Ambrosius P. Snijders, Gavin Kelly, and Frank Uhlmann

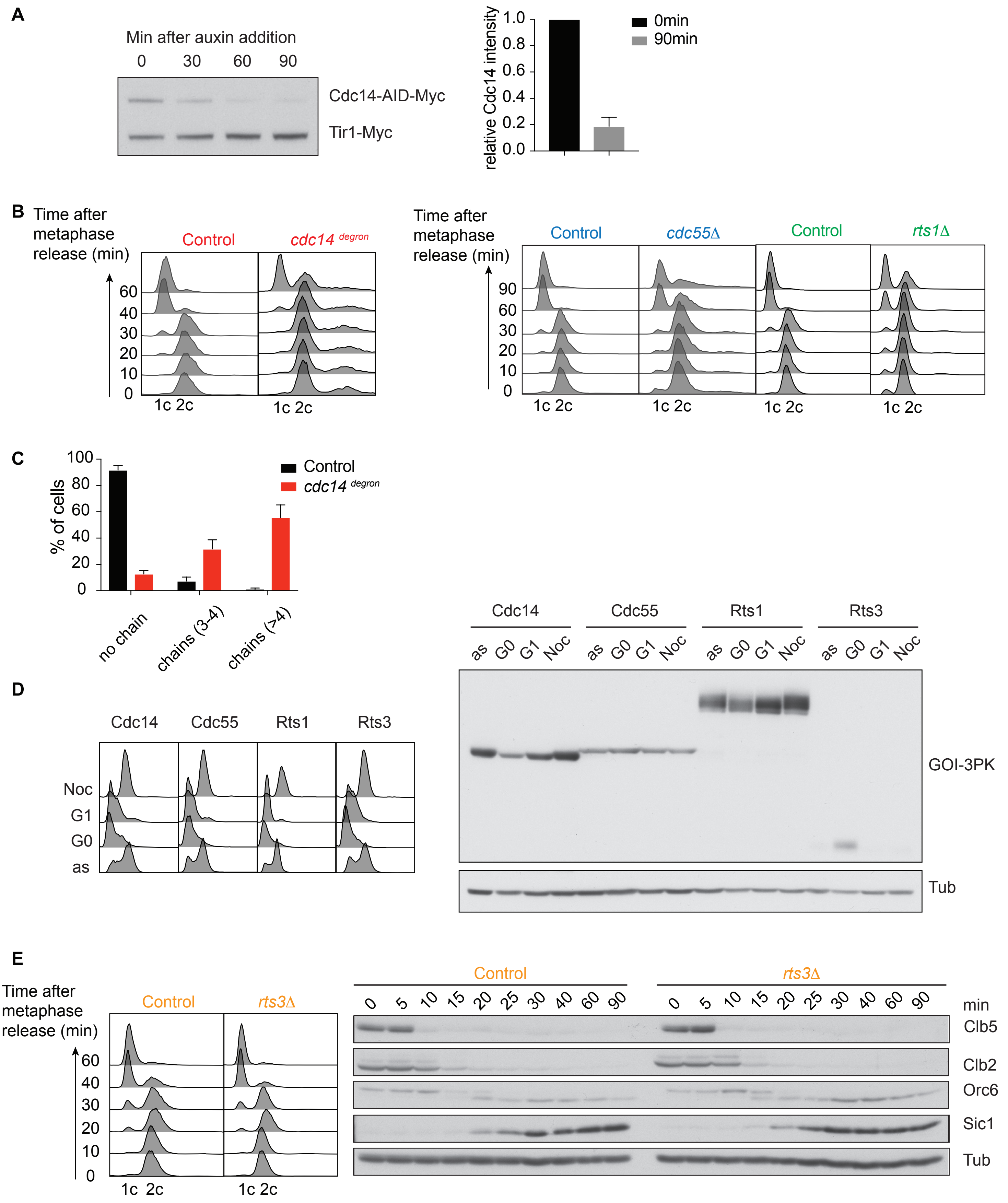

Figure S1. PP2A^{Rts3} Does Not Impact on Mitotic Exit, Related to Figure 1

(A) Cdc14 depletion over time, following auxin addition to a culture of the *cdc14^{degron}* strain, was followed by western blotting (left). Tir1 served as a loading control. Remaining Cdc14 was quantified after 90 minutes (right). The mean and standard deviation from three independent experiments is shown.

(B) FACS analysis of DNA content from a representative experiment shown in Figure 1C.

(C) Cell chain formation was assessed 6 hours following auxin addition to asynchronously proliferating *cdc14^{degron}* cells. The number of connected cell bodies following sonication is depicted. The means and standard deviations from three independent experiments are shown.

(D) Pk epitope-tagged Cdc14, Cdc55, Rts1 or Rts3 was detected in cell extracts from asynchronously growing cells (as), stationary phase cells (G0), following α-factor arrest (G1) and in metaphase arrest following nocodazole (Noc) treatment. Cell cycle stage was confirmed by FACS analysis of DNA content.

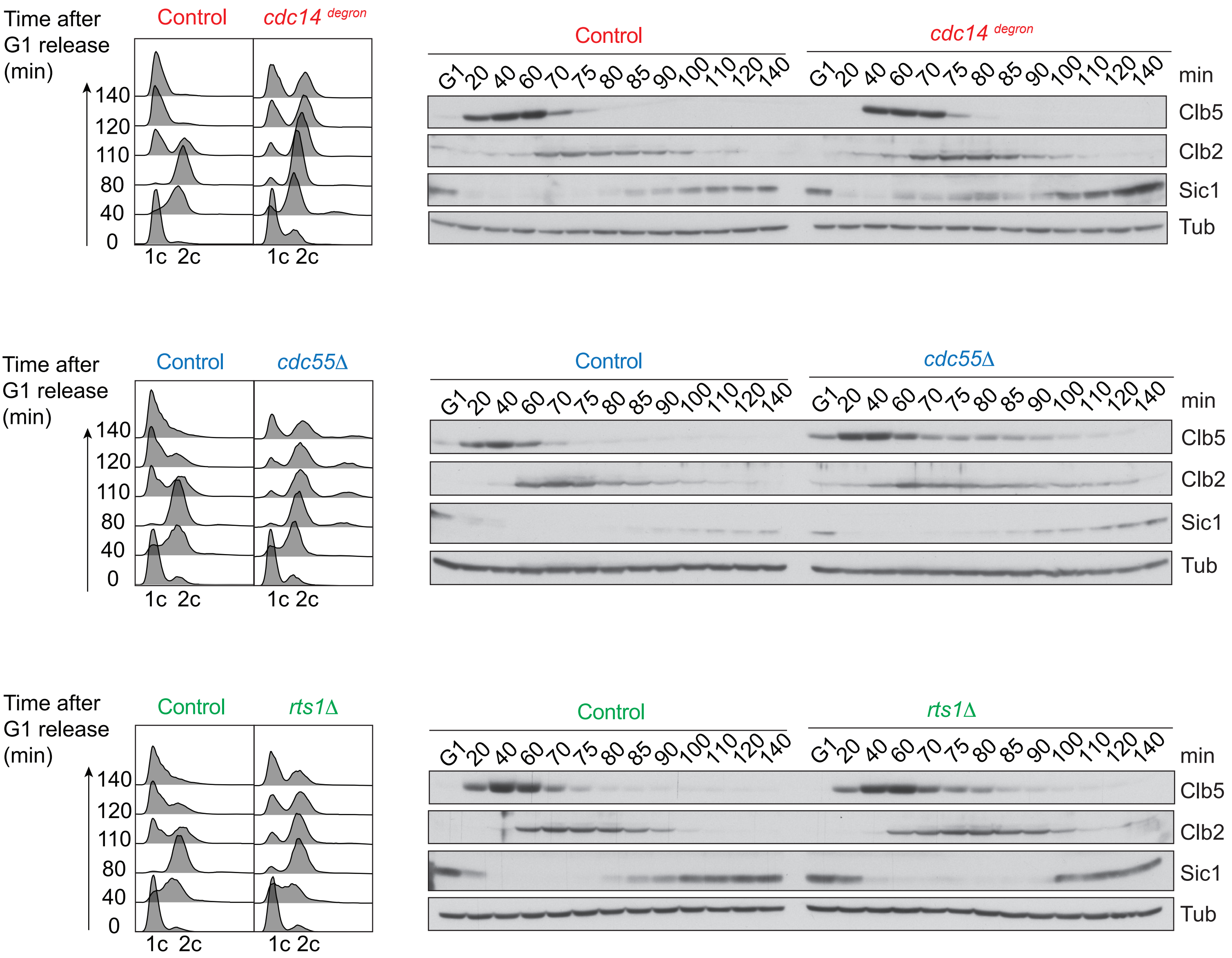

Figure S2. Shared Requirement for Cdc14, PP2A^{Cdc55} and PP2A^{Rts1} for Progression through Mitosis, Related to Figure 1
Control and *cdc14^{degron}* cells were arrested in G1 by α-factor treatment and released to progress synchronously through the cell cycle before being rearrested in G1 by α-factor re-addition. Cell cycle progression was monitored by FACS analysis of DNA content. Protein extracts were prepared at the indicated times and processed for western blotting against the indicated cell cycle proteins. Tubulin served as a loading control. The experiment was repeated with pairs of control and *cdc55*Δ as well as *rts1*Δ cells.

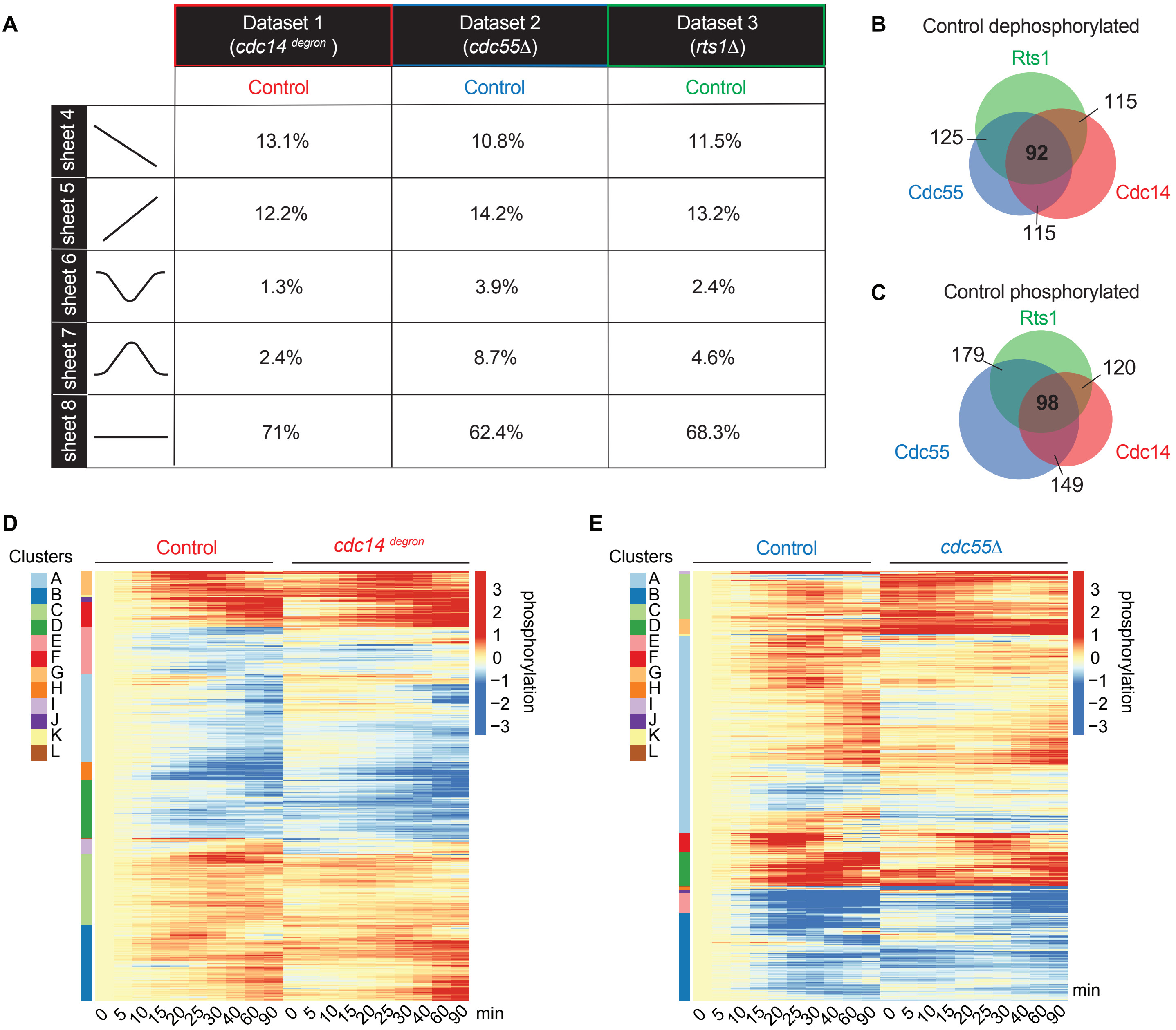

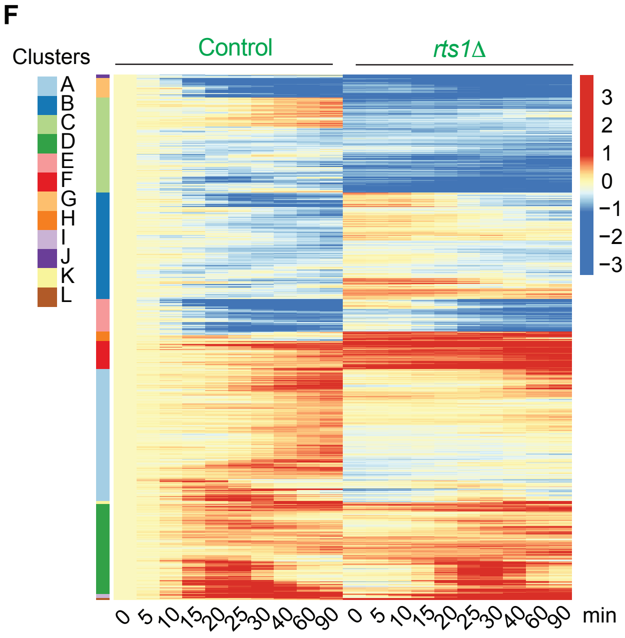

Figure S3. Phosphoproteomics Data Distribution and Heatmap Cluster Analysis, Related to Figure 3

(A) Percentage of phosphosites in each category of each indicated experiment.

(B) Overlap of dephosphorylated phosphosites among the three experimental datasets.

(C) As (B), but overlap of phosphosites that gain in phosphorylation is shown.

(D) Heatmap and cluster analysis of the 691 phosphosites with 1.5 fold phosphorylation changes in either direction in the control strain during mitotic exit in the *cdc14*^{degron} experiment. Phosphorylation abundance in the control strain was normalized to 0 in metaphase (timepoint 0). Rows were ordered by using 12 hierarchical clusters indicated by different colors. Phosphosite increase (red), decrease (blue), and no change (yellow) are depicted. A natural logarithm scale is used. See also Supplemental Dataset 1 for details of the clusters.

(E) As (D), but 1379 phosphosites from the *cdc55*Δ experimental dataset were analyzed. See also Supplemental Dataset 2.

(F) As (D), but 1522 phosphosites from the *rts1*Δ experimental dataset were analyzed. See also Supplemental Dataset 3.

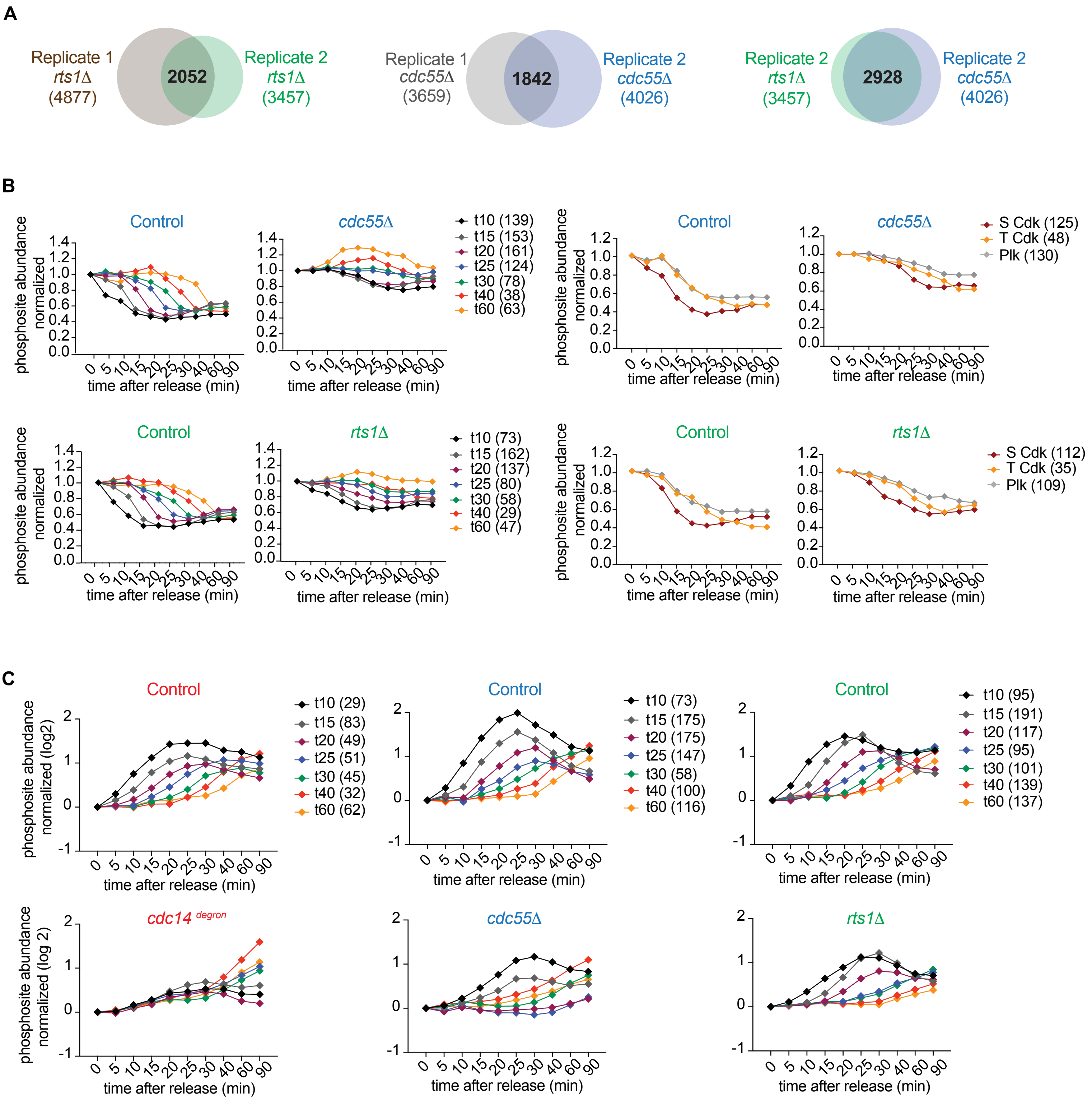

Figure S4. A Biological Replicate and Temporal Order of Phosphorylation Gains During Mitotic Exit, Related to Figure 4

(A) Overlap of phosphosites identified in replicates 1 and 2 of the *cdc55*Δ and *rts1*Δ experimental datasets and between the *cdc55*Δ and *rts1*Δ datasets in replicate 2 which used the same biological control. See also Supplemental Dataset 4.

(B) Normalized mean intensity profiles of phosphosites dephosphorylated during mitotic exit in the control strain of replicate 2, classified according to dephosphorylation timing or kinase consensus motifs, compared to *cdc55*Δ and *rts1*Δ strains as in Figure 4B, C, E and F. See also Supplemental Dataset 4.

(C) Normalized mean intensity profiles of phosphosites that gain abundance during mitotic exit in the control strain in replicate 1. Sites were classified according to the time when their abundance passed the 1.5 fold threshold (top). The same group of phosphosites in the different phosphatase mutant backgrounds are plotted underneath. The number of sites in each group is indicated.

A

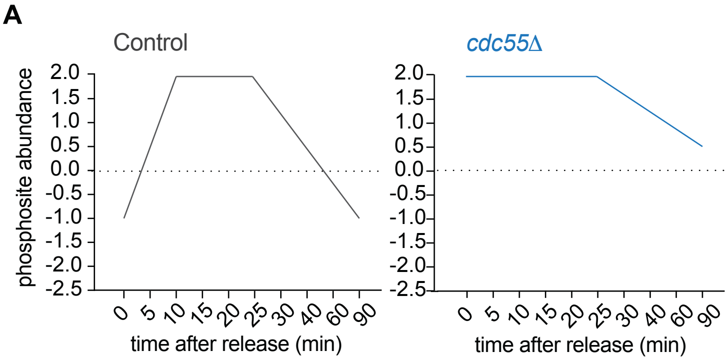

B

Gene names	Positions	Amino acid	Kinase consensus motif
POG1	318	QSANISMANF	Plk
SMI1	355	SQPDTSLQEQ	Plk
YBR138C	241	TKKMVSISDL	
HOF1	424	NQSLSSPSES	Cdk
IDI1	7	TADNNSMPHG	Plk
NET1	288	VDPDKTKQQR	Plk
HOF1	350	QFRNSTPVIR	Cdk / Plk
BUD8	132	PLRNVTPSQN	Cdk / Plk
YCK3	380	KTRNTTPVAT	Cdk / Plk
ADF1	28	KKRSISTAER	
PIB2	346	RKVSETNITN	
NET1	60	RSANVSFTPS	Plk
ERG6	382	TSQEATQ___	Plk
SEC31	1050	QQRVATPLSG	Cdk
PIB2	344	VLRKVSETNI	
KEL1	94	QQRNVSGPST	Plk
KIN1	948	AKREGTPRSI	Cdk / Plk
SYP1	337	LKSKKSSHTL	
NET1	62	ANVSFTPSYF	Cdk
SKG6	167	KYNVRSATPP	
PXL1	44	QRNASTGSLQ	
SPO12	118	QQRFASPTDR	Cdk
TIF4632	880	STKTGSFRNN	
BLI1	109	NNRRQSRTPK	
YCK3	379	SKTRNTTPVA	
ACE2	124	RGLSGTAIFG	
SHE1	287	FSRSTSMNNL	
YMR124W	795	ITSAGSTSGE	
NET1	212	SMRVSTPLAR	Cdk

C

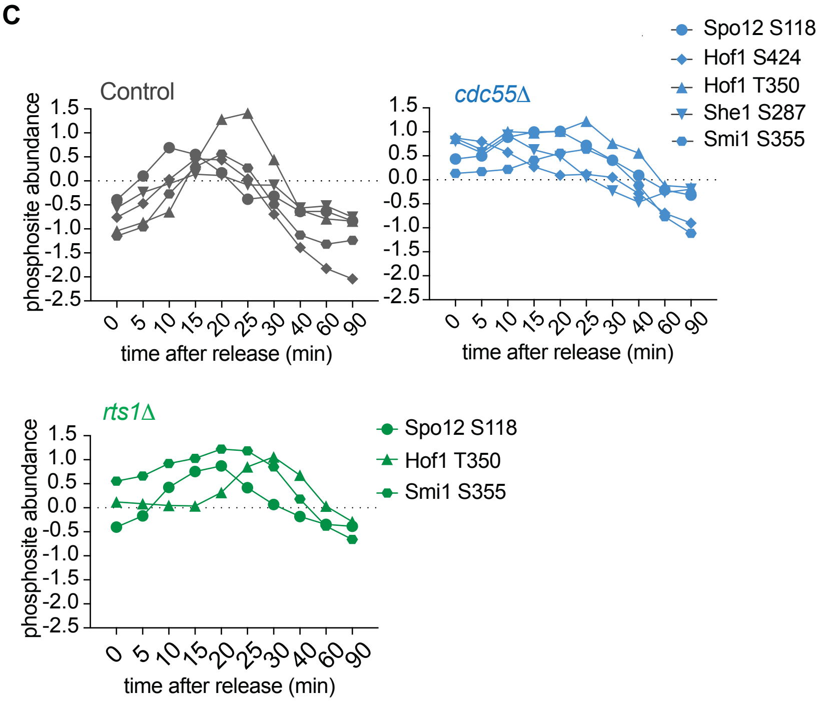

D

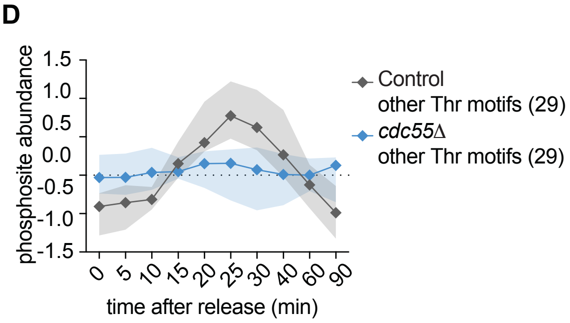

E

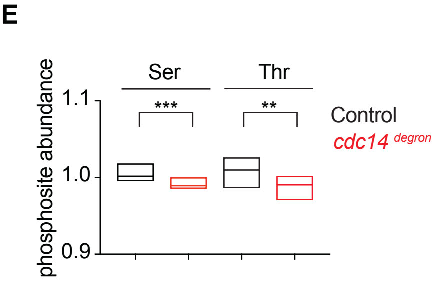

Figure S5. PP2A^{Cdc55} Shapes an Anaphase-Specific Phosphorylation Pattern, Related to Figure 5

(A) Expected profile of an anaphase-specific phosphorylation wave under control of PP2A^{Cdc55}, used as input for correlation analysis.

(B) Table listing phosphosite surroundings and the kinase consensus motif of the 29 identified sites (compare Figure 5C). The phosphorylation site is highlighted in blue.

(C) Example phosphosite profiles of cell cycle substrates controlled by PP2A^{Cdc55} and comparison of lesser control by PP2A^{Rts1}.

(D) Median intensity profile and interquartile range over time of transiently phosphorylated threonine phosphosites that do not adhere to a Cdk or Plk kinase consensus motif.

(E) Median phosphorylation abundance over all 10 time points in control and *cdc14^{degron}* cells, grouped by phosphoacceptor amino acid. 1942 serine and 415 threonine sites entered the analysis. **, p=0.0036; ***, p=0.0006.

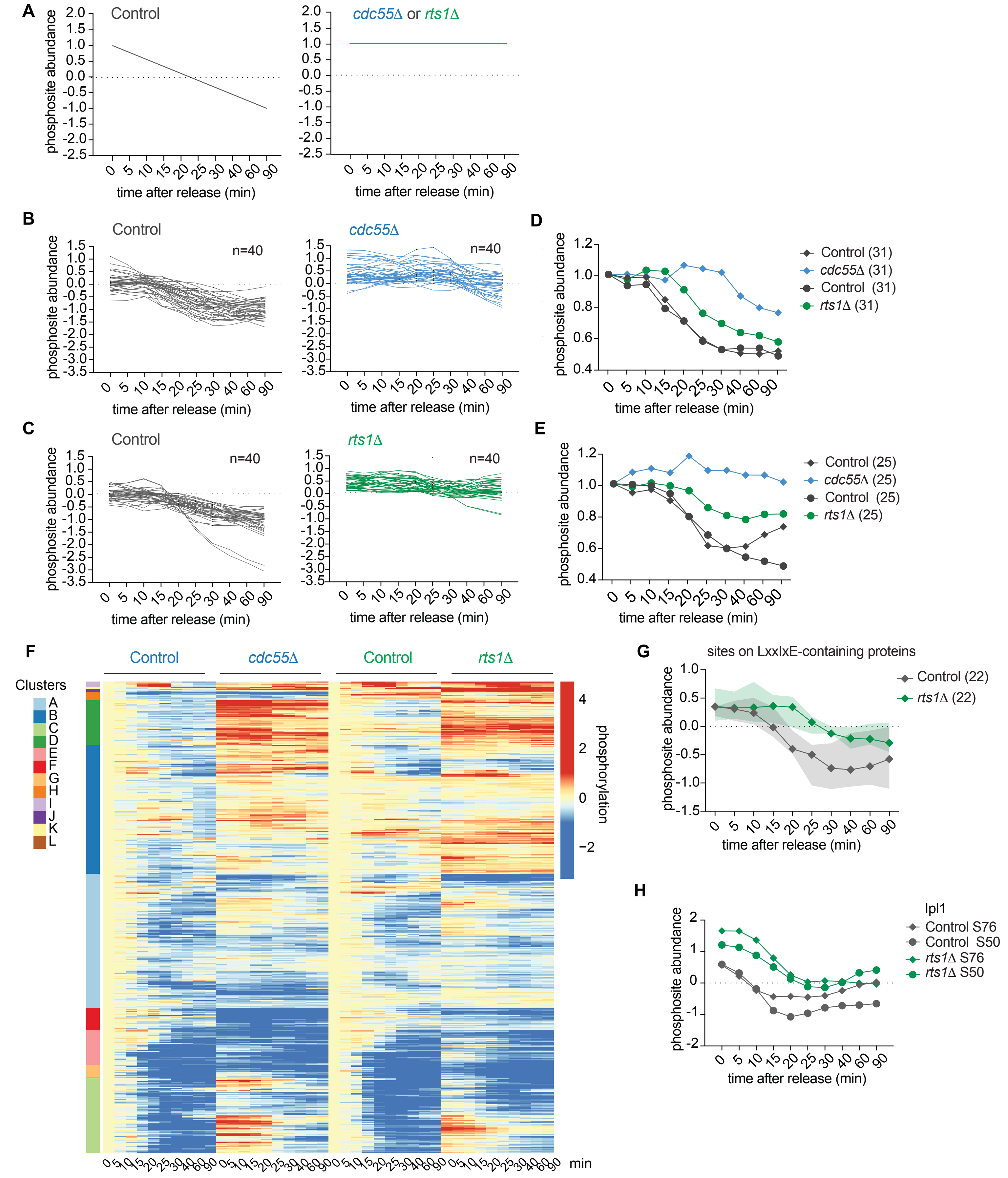

Figure S6. PP2A^{Cdc55} and PP2A^{Rts1} Have Overlapping Substrate Ranges, Related to Figure 6

(A) Expected profiles of PP2A^{Cdc55} or PP2A^{Rts1} substrates, for correlation analysis.

(B) The 40 closest identified PP2A^{Cdc55} substrate profiles. See Supplemental Dataset 2 for a full list of phosphosites.

(C) Normalized median intensity profiles over time of 31 phosphosites in (B) that were also identified in the PP2A^{Rts1} dataset, in control, *cdc55*Δ and *rts1*Δ cells.

(D) The 40 closest identified PP2A^{Rts1} substrate profiles. See Supplemental Dataset 3 for a full list of phosphosites.

(E) Normalized median intensity profiles over time of 25 phosphosites in (D) that were also identified in the PP2A^{Cdc55} dataset, in control, *cdc55*Δ and *rts1*Δ cells.

(F) Heatmap and cluster analysis of the 530 dephosphorylated sites in the control strain in replicate 2 compared to *cdc55*Δ and *rts1*Δ backgrounds, demonstrating related impacts of both phosphatases. See Supplemental Dataset 4 for details of the clusters.

(G) Median intensity profile and interquartile range of 22 phosphosites in 10 proteins harboring a LxxIxE motif in control and PP2A^{Rts1} mutant cells. See Supplemental Dataset 3, sheet 11.

(H) Profile plot of Ipl1 phosphosites in control and *rts1*Δcells.

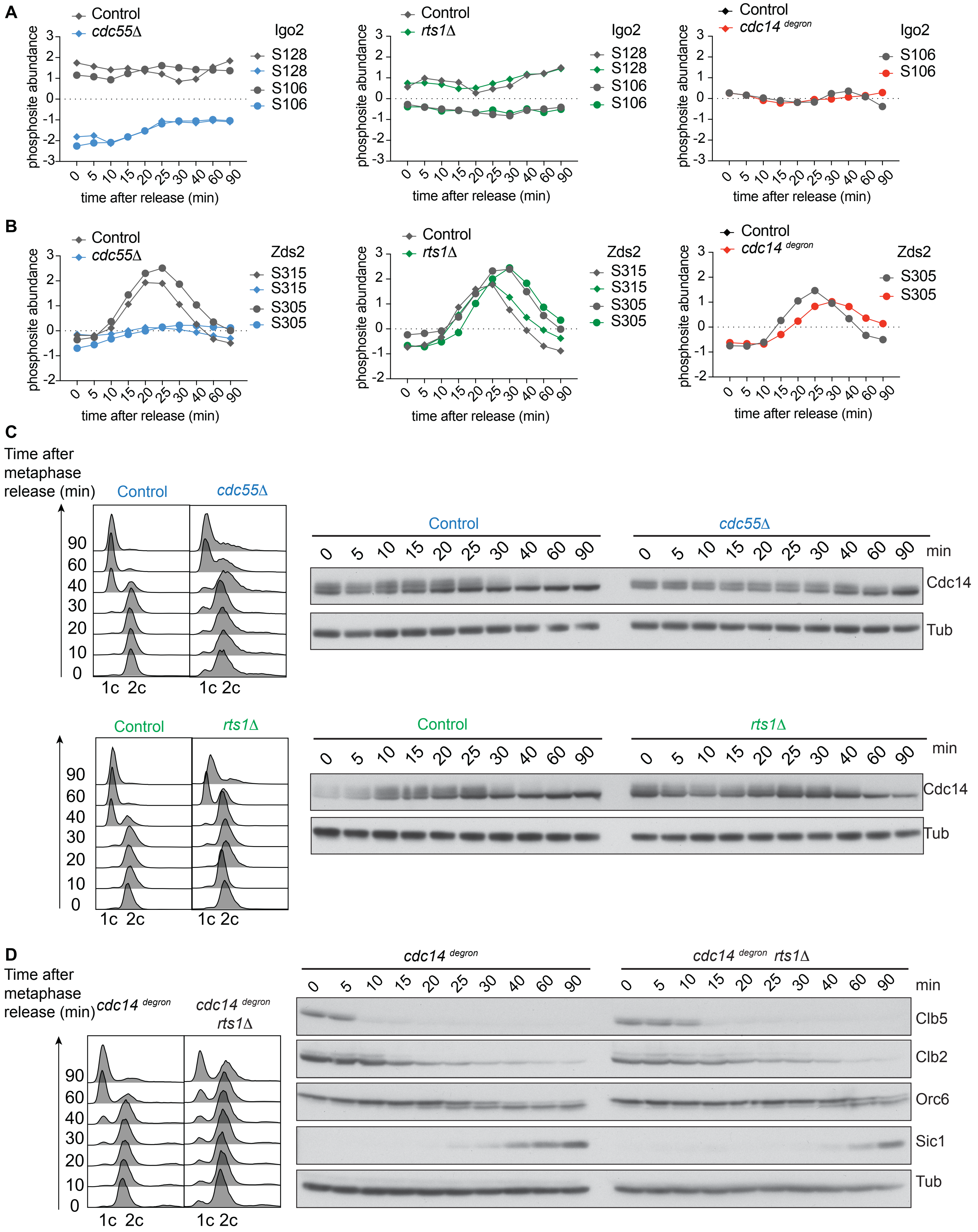

Figure S7. Phosphatase Cooperation during Mitotic Exit, Related to Figure 7

(A) Profile plot of Igo2 phosphosites in control and cdc55Δ cells. Phosphosite profiles are also shown for *rts1Δ* and *cdc14*^{degron} cells.

(B) As (A), but profile plots of Zds2 phosphosites are shown.

(C) Control and cdc55Δ cells (top) or control and *rts1Δ* cells (bottom) were arrested in metaphase and released to progress through mitotic exit as in Figure 1A. Protein extracts were prepared at the indicated times and Cdc14 electrophoretic mobility was monitored by western blotting. FACS analysis of DNA content is shown to confirm synchronous cell cycle progression.

(D) PP2A^{Rts1} acts in synergy with Cdc14. Mitotic exit progression in *cdc14*^{degron} and *cdc14*^{degron} *rts1Δ* cells was compared side-by-side using FACS analysis of DNA content and western blotting against the indicated cell cycle markers. Tubulin served as a loading control.
